# p38α Mitogen-Activated Protein Kinase Is a Druggable Target in Pancreatic Adenocarcinoma

**DOI:** 10.3389/fonc.2019.01294

**Published:** 2019-11-26

**Authors:** Ling Yang, Xiaoting Sun, Ying Ye, Yongtian Lu, Ji Zuo, Wen Liu, Adrian Elcock, Shun Zhu

**Affiliations:** ^1^Department of Cellular and Genetic Medicine, School of Basic Medical Sciences, Fudan University, Shanghai, China; ^2^Department of Medical Oncology, Shuguang Hospital, Shanghai University of Traditional Chinese Medicine, Shanghai, China; ^3^Department of Oral Implantology, Shanghai Engineering Research Center of Tooth Restoration and Regeneration, School and Hospital of Stomatology, Tongji University, Shanghai, China; ^4^Department of ENT, Second People's Hospital of Shenzhen, First Affiliated Hospital of Shenzhen University, Shenzhen, China; ^5^Department of Biochemistry, University of Iowa, Iowa City, IA, United States

**Keywords:** p38α, molecular dynamics, tumor targeted therapy, conformational dynamics, pancreatic cancer

## Abstract

p38 mitogen-activated protein kinases are signaling molecules with major involvement in cancer. A detailed mechanistic understanding of how p38 MAPK family members function is urgently warranted for cancer targeted therapy. The conformational dynamics of the most common member of p38 MAPK family, p38α, are crucial for its function but poorly understood. Here we found that, unlike in other cancer types, p38α is significantly activated in pancreatic adenocarcinoma samples, suggesting its potential for anti-pancreatic cancer therapy. Using a state of the art supercomputer, Anton, long-timescale (39 μs) unbiased molecular dynamics simulations of p38α show that apo p38α has high structural flexibility in six regions, and reveal potential catalysis mechanism involving a “butterfly” motion. Moreover, *in vitro* studies show the low-selectivity of the current p38α inhibitors in both human and mouse pancreatic cancer cell lines, while computational solvent mapping identified 17 novel pockets for drug design. Taken together, our study reveals the conformational dynamics and potentially druggable pockets of p38α, which may potentiate p38α-targeting drug development and benefit pancreatic cancer patients.

## Introduction

p38 mitogen-activated protein kinases (MAPKs) play critical roles in cellular responses, proliferation, survival, cell cycle, and migration in cancer. p38 MAPK family includes p38α (MAPK14), p38β (MAPK11), p38γ (MAPK12), and p38δ (MAPK13). The four p38 MAPK family members have different tissue expression patterns, with p38α being ubiquitously expressed at significant levels in most cell types. The p38 MAPKs function in a cell context-dependent manner (1–4). However, a detailed mechanistic understanding of how p38 MAPK family members function is still not well-understood. A major challenge will be to determine when and how to specifically target p38 MAPK for disease therapy.

To gain insights into its molecular mechanisms and design potential therapeutics, the structure of MAP kinase p38α has been extensively studied over the last two decades (5–8). X-ray crystallography and nuclear magnetic resonance (NMR) showed that p38α consists of two domains, a 135-residue N-terminal domain mainly composed of β-sheets and a 225-residue C-terminal domain mainly composed of α-helices, in between of which lies the catalytic site, i.e., the ATP-binding pocket (9, 10). Despite its seemingly rigid crystal structures, p38α is highly dynamic, which is supported by various evidence. Firstly, in the majority (~78%) of the p38α crystal structures, e.g., 3L8X (11), 3OCG (12), and 2NPQ (13), the glycine-rich loop and/or the activation loop is invisible in the electron density map, indicating the structural flexibility of these loops. Secondly, hydrogen-exchange mass spectrometry (HX/MS) study showed that phosphorylation of the activation loop induced conformational changes in p38α (14); While X-ray crystallographic study showed that phosphorylation brings the N-terminal domain and C-terminal domain closer (15). Thirdly, NMR experiments of p38α in apo and ligand-bound forms suggested that the ATP-binding pocket is highly flexible even after ligand binding (16, 17). These studies reveal the important roles of conformational dynamics in the activation and catalysis of p38α.

Pharmacologically, p38α is considered as a potential target for various diseases such as inflammatory diseases and cancer. The potent small-molecule inhibitor SB203580 competitively binds the ATP-binding pocket and has been widely used to study p38 MAPK functions (5). Nevertheless, given the similarity of the ATP-binding site in different kinases, SB203580 also targets non-p38 protein kinases, usually at higher concentrations (18). Over the years, many p38α inhibitors have been developed, for example BIRB796, which leads to a conformational reorganization that prevents ATP binding and activation (19). The C-terminal domain has also been predicted to have the flexibility for potentially binding differently shaped compounds (20). For p38α inhibitor development, allosteric small-molecule inhibitors that target other regions of the kinase are warranted and they might reduce the off-target effects in drug applications.

Clinically, p38 MAPK inhibitors show significant effects in pre-clinical animal models but repeatedly failed in clinical trials (21). In cancer study, p38α and p38β increase cell proliferation and invasion of colon cancer, follicular lymphoma, ovarian cancer, and more recently, pancreatic cancer (22–25). It is indicated that targeting p38 MAPKs, especially p38α, should be explored for cancer therapy. However, several phase I clinical studies with p38 MAPK small-molecule direct or indirect inhibitors show hepatic, neurological, gastrointestinal, and cardiovascular toxicities, indicating that the commonly studied inhibitors are not highly selective (26). Currently, one of the major challenges in pancreatic cancer drug development is to overcome drug off-target effects and drug resistance (26, 27). It is rational to speculate that next-generation highly selective p38 MAPK inhibitors may exhibit less adverse effects. However, it still requires further investigation in cancer patients.

In this work, by screening pancreatic adenocarcinoma (PDAC) patient samples (*n* = 40) and TCGA database, we demonstrated that p38 MAPKs, especially p38α are highly expressed and activated. p38α blockades show significant anti-tumor effect in PDAC cells, but are not selective enough. To pave the way for highly selective inhibitor development, conformational dynamics of p38α was examined. Anton supercomputer long-timescale (39 μs) MD simulations using both AMBER and OPLS force fields show the p38α flexibility in six regions. Mechanistically, p38α MD simulations reveal a “butterfly” motion that might be important for p38α catalytic function. In addition, computational solvent mapping reveals 17 novel pockets that are potentially druggable for cancer therapy. To our knowledge, this is the first comprehensive study of both the conformational dynamics and potentially druggable pockets in p38α. This study provides insights into understanding the molecular mechanism of p38α function and into developing potential drugs with high specificity and selectivity against PDAC.

## Results

### p38 MAPKs Expression Correlates With Poor Prognosis in PDAC Patients

**Figure 1:**
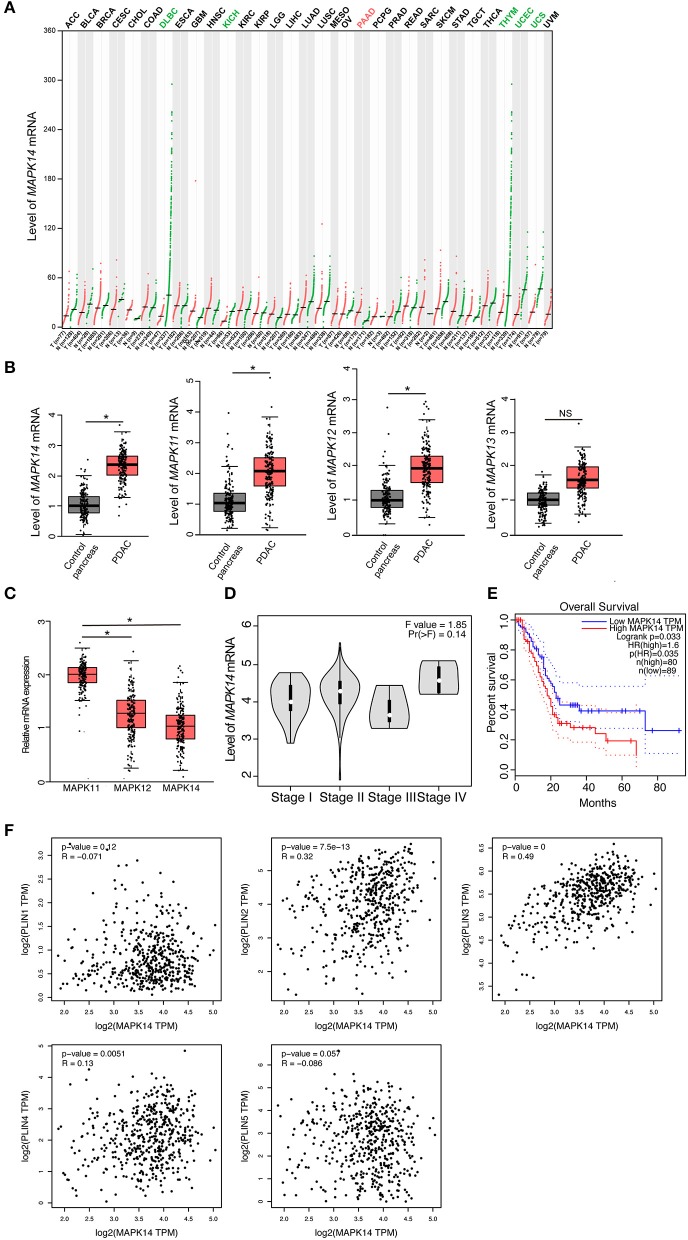
p38α expression correlates with poor prognosis and adipose markers in PDAC patients. **(A)** Transcriptomic expression levels of *MAPK14* across multiple cancer types and paired normal samples, with each dot representing a distinct tumor or normal sample. Red dot, tumor sample; Green dot, control sample; Red group name, significantly upregulated; Green group name, significantly downregulated; Black group name, not significant. **(B)** Transcriptomic expression levels of *MAPK14, MAPK11, MAPK12*, and *MAPK13* in PDAC and adjacent pancreas samples [n(Control) = 179 samples; *n* (PAAD) = 171 samples]. **(C)** Transcriptomic expression levels of *MAPK14, MAPK11* and *MAPK12* in PDAC samples [*n* (PAAD) = 171 samples]. **(D)** Violin plots of *MAPK14* based on PDAC patient pathological stage [*n* (PAAD) = 171 samples]. **(E)** Overall survival (OS) analysis of PDAC patients based on *MAPK14* expression. **(F)** Correlation analysis of *MAPK14* and adipose markers (*PLIN1, PLIN2, PLIN3, PLIN4, PLIN5*) expression in human PDAC tissues and adjacent pancreas. Control group = 179 samples; PAAD group = 171 samples. *p < 0.05. NS = not significant. Data presented as mean ± s.e.m.

To investigate the role of p38 MAPKs in cancer, we screened a panel of human tumor tissues in The Cancer Genome Atlas (TCGA) that spontaneously express p38 MAPKs. We have found that the majority of the tumor tissues express similar level of p38 MAPKs to its adjacent healthy tissues (black labeled, Figure 1A, Figures S1A–C), while uterine carcinosarcoma (UCS), uterine corpus endometrial carcinoma (UCEC), and chromophobe renal cell carcinoma (KICH) show decreased *p38*α (*MAPK14*), *p38*β (*MAPK11*), and *p38*γ (*MAPK12*) expression compared with control pancreas (green labeled, Figure 1A, Figures S1A,B). Surprisingly, between more than 30 types of cancer, only pancreatic adenocarcinoma (PAAD or PDAC) shows a significant increase of *p38*α, *p38*β, and *p38*γ compared with healthy control pancreas (red labeled, Figure 1A, Figures S1A,B), suggesting that p38 MAPKs may be involved in PDAC development. To further validate these findings, we detected the mRNA level for all four members of the p38 MAPK family. Indeed, *p38*α, *p38*β, and *p38*γ are all increased markedly, while *p38*δ (*MAPK13*) shows a trend of increase (Figure 1B). *p38*α shows a significant dominance among these genes (Figure 1C), indicating its importance among four members. We next investigated the relationship of p38 with PDAC clinical outcomes. Surprisingly, *p38*α showed a trend of increasing with advanced stage, and strongly correlated with poor overall survival in PDAC patients (Figures 1D,E). These results suggest that p38α plays a role in PDAC progression and correlates with poor prognosis in PDAC patients.

### p38α Expression Correlates With Adipose Markers in PDAC Tissues

It was reported that proliferating cancer cells may take up exogenous lipids and activating endogenous lipid biosynthesis (28), and tumor implanted in adipose environment show significant lipid metabolic reprogramming (29). Considering that PDAC is one of the tumors that adjacent to the adipose environment, we tested the correlation of p38 MAPKs and lipid droplet marker perilipin (PLIN) family in the PDAC database. Surprisingly, *p38*α strongly correlated with PLIN 2 and 3, two small lipid droplets markers, but not PLIN1, 4, and 5, which are the big lipid droplets marker (Figure 1F). It was plausible that small lipid droplets exist inside pancreatic tumor cells or in mobilized adipocytes in the tumor microenvironment. These findings support that *p38*α correlates with adipose-rich PDAC and may be involved in cancer lipid metabolism.

### p38α Is Activated in PDAC Patient Samples

**Figure 2:**
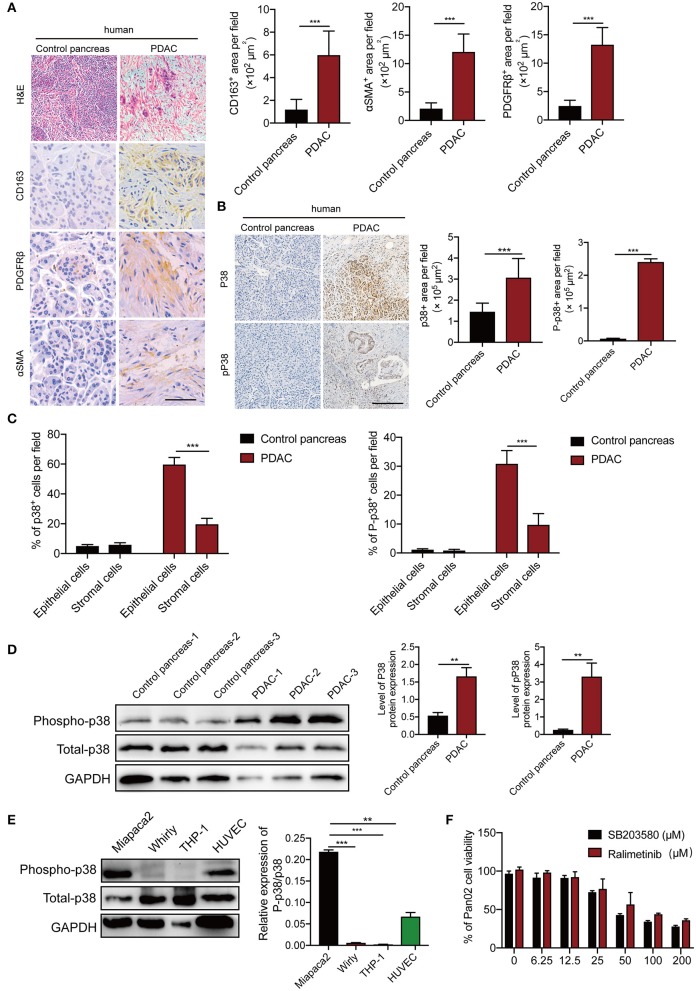
p38α is activated in cancer cells in PDAC patient samples. **(A)** Micrographs of H&E and immunohistochemistry staining with CD163, PDGFRβ, and αSMA. Quantification of CD163^+^, PDGFRβ^+^, and αSMA^+^ signals (*n* = 8 random fields per group). **(B)** Micrographs of immunohistochemistry staining with p38 and Phospho-p38 in PDAC and adjacent pancreas samples. Quantification of p38^+^, Phospho-p38^+^ signals (*n* = 8 random fields per group). **(C)** Pathological analysis of p38 and Phospho-p38 in PDAC and adjacent pancreas samples (*n* = 20 samples per group). **(D)** Protein expression levels of p38 and Phospho-p38 in human PDAC tissues and adjacent pancreas (*n* = 20 samples per group). **(E)** protein expression levels of p38 and Phospho-p38 in various human cell lines (*n* = 3 samples per group). **(F)** Cell viabilities of Pan02 cell lines treated with 3.125–200 μM SB203580 for 24 h (*n* = 6 samples per group). *p < 0.05; **p < 0.01; ***p < 0.001. NS = not significant. Data presented as mean ± s.e.m.

To validate our findings, we measured p38α levels in human PDAC patients. In this study, the patient PDAC sample and adjacent pancreas were compared. In accordance with previous report (30), PDAC tissues show significant infiltration of inflammatory cells, stromal cellular components, and activated fibroblasts (Figure 2A). Next, we detected p38α in these samples. As expected, p38α is highly expressed in human PDAC tissues (Figures 2B,D; Figure S2). Consistent with this result, western blot and immunohistochemistry staining show highly activated phospho-p38α in human PDAC tissues (Figures 2B,D). Furthermore, pathological analysis shows that both p38α and phospho-p38α are mainly located in epithelial cancer cells (Figure 2C). Western blot of various cell lines demonstrates that PDAC cancer cells and endothelial primary cells expressed high levels of p38α protein, whereas human THP-1 macrophage-like cell line, and human stromal fibroblasts lacked p38α expression (Figure 2E). These findings further support our notion that p38α is activated in PDAC cells. These pilot clinical findings validate our TCGA data.

### p38α Blockade Enhances Apoptosis of Human and Mouse PDAC Cells

To gain further mechanistic insights of p38α blockade on cancer, human pancreatic adenocarcinoma cells MiaPaca-2, Panc-1, and mouse PDAC cell line Panc02 were projected for p38α blockade. The canonical inhibitor SB203580, which can block both p38α and p38β (31), and ralimetinib, a small molecule inhibitor specifically for p38α under phase 2 clinical trial (32), were tested. As expected, both drugs significantly inhibit PDAC cell viability, with the IC50 around 56.89μM (Figure 2F). These findings suggest the potential of p38α as a therapeutic target for PDAC.

### p38α Is Highly Dynamic Locally and Globally

**Figure 3:**
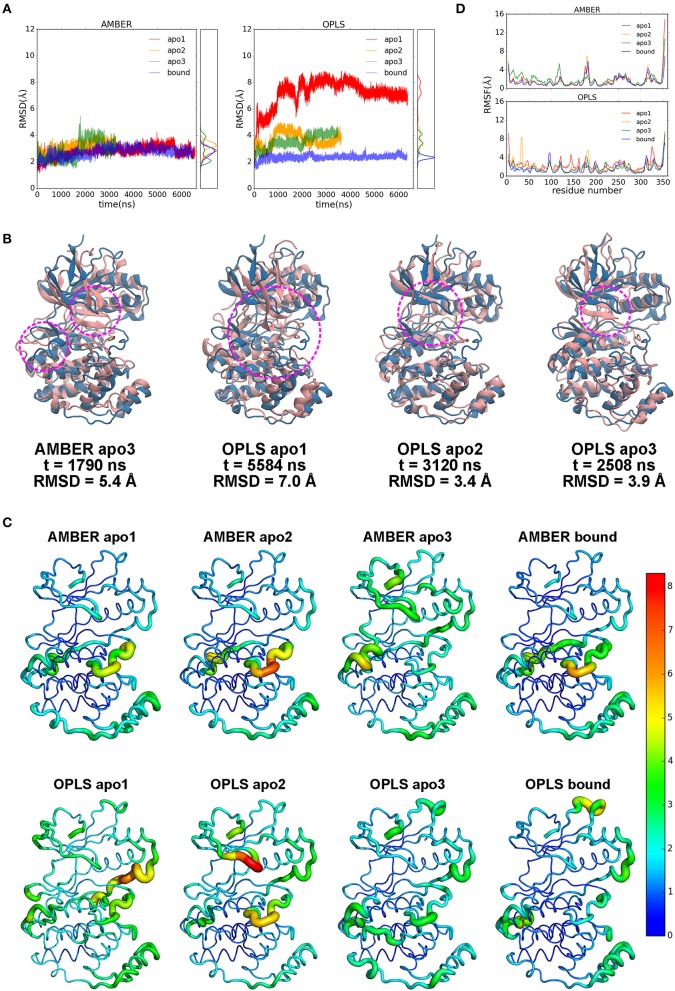
Dynamics of p38α kinase in MD simulations. **(A)** RMSDs of p38α in AMBER and OPLS simulations. Three runs of apo p38α (apo1, apo2, and apo3) and one run of ligand-bound p38α are shown in red, orange, green, and blue, respectively. The probability density functions are shown in the right panel. **(B)** Representative snapshots (pink) aligned to the initial structure (light blue). Regions of interest are marked by magenta circle. **(C)** RMSFs mapped onto p38α structure. Sausage representation is used for the protein. The thickness of the tube is normalized within each simulation run with thicker tubes corresponding to higher RMSFs. The color of the tube is normalized using all simulation runs using the “rainbow” gradient with warmer color corresponding to higher RMSFs. Color normalization was done by setting the color range to 0–8.3 Å (the largest RMSF value in all simulations). The N-terminal end (residue 4–13) and C-terminal end (residue 345–354) are not shown and excluded from the color normalization for better visualization. **(D)** RMSFs of p38α backbone in AMBER and OPLS simulations. The color scheme is same as in RMSD.

Despite its potential for PDAC therapy, our understanding of the structural and functional basis of p38α is still poor. To investigate the structural dynamics of p38α, we applied the state of the art supercomputer, Anton for simulation of our potential targets (33). Three simulation runs of apo p38α and one simulation run of ligand-bound p38α (Table 1) for the AMBERff99SB-ILDN and OPLS-AA/L force fields (abbreviated as AMBER and OPLS) were performed (Table 2) and the convergence was evaluated (Figure S3). We plot the root mean square deviations (RMSDs) of the protein backbone from their crystallographic positions for all simulation runs. In AMBER simulations, RMSDs of apo p38α range from 2 to 5 Å (Figure 3A, left panel, red, orange, and green lines); in OPLS simulations, RMSDs of the apo p38α run go up to 8 Å (Figure 3A, right panel, red line). In contrast, ligand-bound p38α is below 4 Å in both AMBER and OPLS simulations (Figure 3A, blue lines). The RMSD discrepancy between AMBER and OPLS simulations is possibly due to their different preference for secondary structure propensities. The RMSD discrepancy between apo and ligand-bound p38α suggests that the conformational flexibility of p38α is dampened upon ligand binding. It should be noted that as the N terminal and C terminal ends of p38α can be quite floppy (Figure S4), these ends were truncated in MD simulations. These results show that apo p38α exhibits higher structural flexibility than ligand-bound p38α.

**Table 1:** Equilibrium angles for the eight restrained dihedral angles of the ligand SB203580.

**a_**i**_-a_**j**_-a_**k**_-a_**l**_**	**φ(**°**)**
CB5-CB4-CC5-CC4	42.99
CB5-CB4-CC5-NC1	−138.65
CB3-CB4-CC5-CC4	−136.12
CB3-CB4-CC5-NC1	42.23
CD1-CD6-CC4-NC3	56.05
CD1-CD6-CC4-CC5	−124.05
CD5-CD6-CC4-NC3	−122.52
CD5-CD6-CC4-CC5	57.38

*The atom names are consistent with the crystal structure (PDB code: 1A9U)*.

**Table 2:** Simulation parameters for MD simulations of apo and SB203580-bound p38α.

**Type**	**Form**	**Force field**	**Box size (Å^**3**^)**	**#Atoms**	**Duration (μs)**
Solution	Apo	AMBER	85.0 × 85.0 × 85.0	60,120	6.5(apo1); 3.2(apo2); 3.2(apo3)
Solution	Bound	AMBER	85.0 × 85.0 × 85.0	60,253	6.4
Solution	Apo	OPLS	85.0 × 85.0 × 85.0	60,120	6.3(apo1); 3.6(apo2); 3.4(apo3)
Solution	Bound	OPLS	85.0 × 85.0 × 85.0	60,253	6.3

To show global structural excursions of apo p38α, we visually inspected trajectories of simulation runs and found several representative conformations (Figure 3B, pink ribbons) that were aligned to the crystal structure (Figure 3B, light blue ribbons). In AMBER apo3, we see a spike in the RMSD plot at ~1,790 ns (Figure 3A, left panel, green line), which attributes to the loss of helicity in αD helix and “closing” motion of the glycine-rich loop (Figure 3B, AMBER apo3, magenta circles; Figure S5). In OPLS apo1, RMSD rises rapidly to ~8 Å (Figure 3A, right panel, red line), which can be mainly attributable to the “closing” motion of the glycine-rich loop that is accompanied by large conformational changes in both the N-terminal and C-terminal domains (Figure 3B, OPLS apo1 magenta circle). In OPLS apo2, RMSD climbs up to ~4.5 Å at ~1,000 ns and descends to 3.5 Å at ~2,000 ns (Figure 3A, right panel, orange line), which identify the “closing” and “opening” of the glycine-rich loop (Figure 3B, OPLS apo2, magenta circle). In OPLS apo3, RMSD rises steadily to ~4 Å (Figure 3A, right panel, green line), which classifies the “closing” of the glycine-rich loop (Figure 3B, OPLS apo3, magenta circle). Among these structural excursions, OPLS apo1 seems to have the highest RMSD at 7.0 Å (Figure 3B, OPLS apo1, magenta circle), probably due to the global conformational change of both the N-terminal and C-terminal domains. It is also intriguing to see that in both OPLS apo1 and OPLS apo2, the glycine-rich loop is deformed significantly compared with the β-hairpin structure in the crystal structure (Figure 3B, OPLS apo1 and OPLS apo2).

Furthermore, to understand the local structural dynamics of p38α, residue-based root mean square fluctuations (RMSFs) were calculated and mapped onto the p38α structure (Figure 3C). In general, we found that local structural dynamics is highly dependent on the secondary structure of the local region. In α-helices- or β-sheets-rich local regions, low structural flexibility is observed, such as β-sheets 1–5 in the N-terminal domain (Figure S6) (34), and C-terminal domain that is made up of α-helices E, F, H, and G (Figure 3C, blue and cyan tubes). In local regions mainly consisting of disordered secondary structures such as loops and turns, high structural flexibility is observed (Figure 3C, orange and red tubes). In AMBER and OPLS simulations of p38α, high structural flexibility is observed mainly in 6 local regions (Figures 3C,D): (1) the glycine-rich loop (residue 30–38); (2) the L6 loop (residue 93–99); (3) the αD helix (residue 113–119); (4) the activation loop (residue 169–183); (5) the MAP kinase insert (residue 243–261); (6) the L16 loop (residue 305–330). Taken together, these data suggested p38α is highly dynamic in the entire kinase structure and in the specific secondary structures of local regions.

### “Butterfly” Motions Potentially Contribute to p38α Enzymatic Catalysis

**Figure 4:**
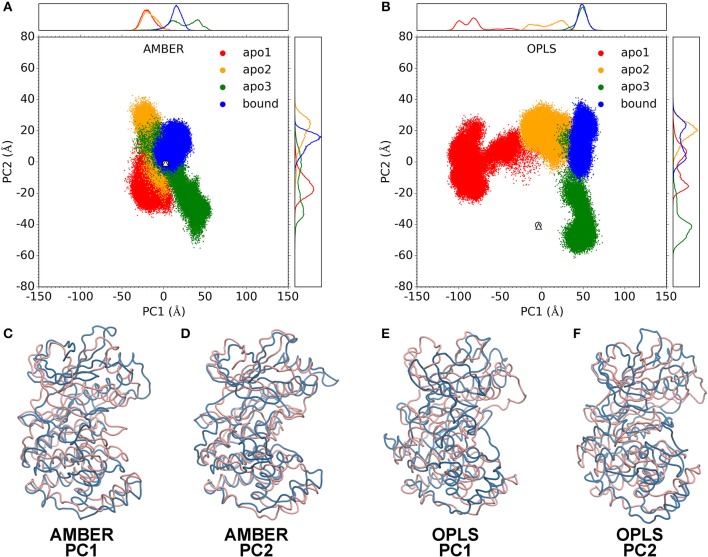
Principal component analysis. PC1-PC2 plots for AMBER simulations **(A)** and OPLS simulations **(B)**. Data for apo1, apo2, apo3 and bound simulations are shown as red, orange, green, and blue dots. Data for the crystal structures of apo p38α (PDB code: 1P38) and SB203580-bound p38α (PDB code: 1A9U) are shown as white circle and triangle, respectively on the PC1-PC2 plot. Probability density functions are shown in upper and right panels. **(C–F)** show the two extreme structures along the PC1 or PC2 axis as blue and pink ribbons. Blue and pink indicate the – and + axis, respectively.

To gain further insights on the catalysis mechanism of p38α, we performed a principal component analysis (PCA) on p38α trajectories. For both AMBER and OPLS simulations, the trajectories of three apo p38α runs and one ligand-bound p38α run were combined to build a structure ensemble for PCA. The two most dominant PCs, PC1 and PC2, account for a large fraction of the correlated motions (**S7A**). Both AMBER and OPLS force fields produce considerable structural excursions (as big as 100 Å) from the crystal structures (Figures 4A,B). AMBER simulations show a more restricted structural excursion pattern (Figure 4A) while OPLS simulations have a more expanded pattern (Figure 4B). These results are in accordance with our previous findings (Figure 3A).

Structural morphing between the two extreme structures along the PC1 and PC2 axis was performed (Movies S1–S4). For PC1 and PC2, we overlaid the two extreme structures onto each other (Figures 4C–F). Note that the pink and blue structures represent the + and – axis. In AMBER simulations, PC1 represents the “butterfly” motion between the N-terminal and C-terminal domains (Figure 4C; Movie S1), where the N-terminal domain and the C-terminal domain resembling the wings of “butterfly”; PC2 represents the “twist” motion, where the N-terminal domain and the C-terminal domain rotates relative to each other (Figure 4D; Movie S2). In OPLS simulations, PC1 represents a large conformational change where both the N-terminal domain and the C-terminal domain move toward to each other, completely occluding the ATP-binding pocket (Figure 4E; Movie S3); PC2 represents a “butterfly” motion (Figure 4F; Movie S4). Notably, although the similarity of PCs in AMBER and OPLS simulations are limited (Figure S7B), it appears that both force fields have a reasonable agreement with the experimental crystal structures (Figures S7C,D).

### Simulations Agree With Experimental NMR Observables

**Figure 5:**
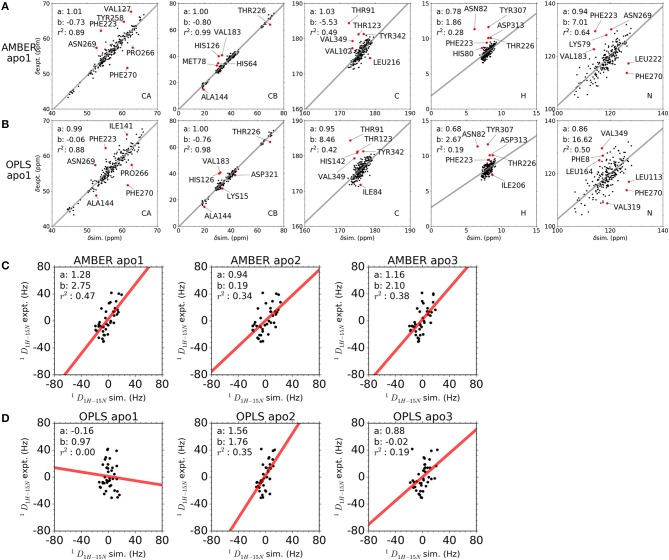
Comparison between simulated and experimental NMR observables. **(A,B)** Comparison between simulated and experimental chemical shifts of apo p38α for simulation runs AMBER apo1 and OPLS apo1. Chemical shifts were calculated from MD snapshots at an interval of 1 ns using PPM. The x-axis and y-axis of the correlation plot are the simulated and experimental chemical shifts, respectively. The linear regression lines are shown as gray, with slope a, intersection b, and correlation coefficient *r*^2^ shown in top left corner. The top six outlier residues with the largest RMS error are shown as red dots. **(C,D)** Comparison between simulated and experimental RDCs of apo p38α. Results are shown for all AMBER simulations and OPLS simulations of apo p38α. The x-axis and y-axis of the correlation plot are the simulated and experimental RDCs, respectively. The linear regression lines are shown as red, with slope a, intersection b, and correlation coefficient *r*^2^ shown in top left corner.

Chemical shifts have been important indicators of local backbone conformations (35). For verification of our results, we compared simulated and experimental chemical shifts of apo p38α. Linear regression of chemical shifts for five atom types in two representative simulation runs is performed (Figures 5A,B). The correlation coefficient (*r*^2^) suggest that there is excellent agreement for atom CB (*r*^2^: 0.98, 0.99) and atom CA (*r*^2^: 0.88, 0.89); reasonable agreement for atom C and N (*r*^2^: 0.42, 0.64); and bad agreement for atom H (*r*^2^: 0.19, 0.28). The correlation coefficient of the five atoms ranks similarly in other simulation runs (Figure S8). The top six outlier amino acid residues were highlighted (Figures 5A,B, red dots). To confirm the PPM results, we also performed chemical shift predictions using SHIFTX2. As expected, similar results were observed, indicating the accuracy of our simulation data (Figures S9–S11). Despite different structural flexibility in AMBER and OPLS simulations, there seems to be no significant difference between the accuracy of the chemical shifts. Furthermore, experimental residual dipolar couplings (RDCs) of apo p38α were applied for accuracy validation (Table 3). We compare simulated and experimental RDCs for 39 residues (Figure S12A) in apo p38α (36). The probability density function of Pearson's R (between simulated and experimental RDCs) indicated that MD simulations using both force fields agree generally well with RDC experiments (Figure S12B). Linear regressions of simulated and experimental RDCs show the same trend except OPLS apo1 (*r*^2^ is 0.00), possibly due to the surface charge distribution difference for all 85 charged residues (Table 4, Figure S13). The other five simulation runs agree generally well (*r*^2^ is 0.47, 0.34, 0.38, 0.35, 0.19 for AMBER apo1, AMBER apo2, AMBER apo3, OPLS apo2, OPLS apo3; Figures 5C,D). Taken together, these data suggest the high quality and accuracy of our simulations.

**Table 3:** Experimental ^1^H–^15^N residual dipolar couplings of the 39 residues in apo p38α.

**Resid**	**Residue**	**RDC (Hz)**
17	ILE	−21.25
19	GLU	4.117647
20	VAL	13.71324
23	ARG	−2.61029
27	LEU	−7.79412
28	SER	40.40441
33	GLY	−9.11765
36	GLY	−0.29412
42	PHE	18.23529
43	ASP	−14.9632
56	SER	−7.79412
57	ARG	−1.94853
60	GLN	−15.8456
61	SER	−12.5368
81	GLU	38.75
91	THR	−6.47059
93	ALA	−13.75
94	ARG	18.56618
97	GLU	−4.70588
98	GLU	9.522059
99	PHE	12.83088
101	ASP	−8.18015
129	PHE	−2.83088
296	ARG	−21.1397
297	ILE	16.13971
298	THR	29.15441
300	ALA	−30.9559
306	ALA	−5.42279
309	ALA	−25.1654
310	GLN	7.647059
317	GLU	41.39706
319	VAL	13.60294
321	ASP	26.17647
330	ARG	−20.1471
331	ASP	27.44485
332	LEU	−9.33824
333	LEU	13.82353
335	ASP	−30.3493
343	ASP	−26.5441

*Data were extracted from the reference Honndorf et al. (36)*.

**Table 4:** List of charged residues used in distance matrix calculations.

**Index**	**Residue**
0	GLU4
1	ARG5
2	ARG10
3	GLU12
4	LYS15
5	GLU19
6	GLU22
7	ARG23
8	ASP43
9	LYS45
10	ARG49
11	LYS53
12	LYS54
13	ARG57
14	LYS66
15	ARG67
16	ARG70
17	GLU71
18	ARG73
19	LYS76
20	LYS79
21	GLU81
22	ASP88
23	ARG94
24	GLU97
25	GLU98
26	ASP101
27	ASP112
28	LYS118
29	LYS121
30	ASP124
31	ASP125
32	ARG136
33	LYS139
34	ASP145
35	ARG149
36	ASP150
37	LYS152
38	GLU160
39	ASP161
40	GLU163
41	LYS165
42	ASP168
43	ARG173
44	ASP176
45	ASP177
46	GLU178
47	ARG186
48	ARG189
49	GLU192
50	ASP205
51	GLU215
52	ARG220
53	ASP227
54	ASP230
55	LYS233
56	ARG237
57	GLU245
58	LYS248
59	LYS249
60	GLU253
61	ARG256
62	LYS267
63	ASP283
64	GLU286
65	LYS287
66	ASP292
67	ASP294
68	LYS295
69	ARG296
70	ASP313
71	ASP315
72	ASP316
73	GLU317
74	ASP321
75	ASP324
76	GLU328
77	ARG330
78	ASP331
79	ASP335
80	GLU336
81	LYS338
82	ASP343
83	GLU344
84	ASP354

*Indices are consistent with that in distance matrix plots*.

### Dynamics of p38α Inhibitor in the ATP-Binding Pocket

**Figure 6:**
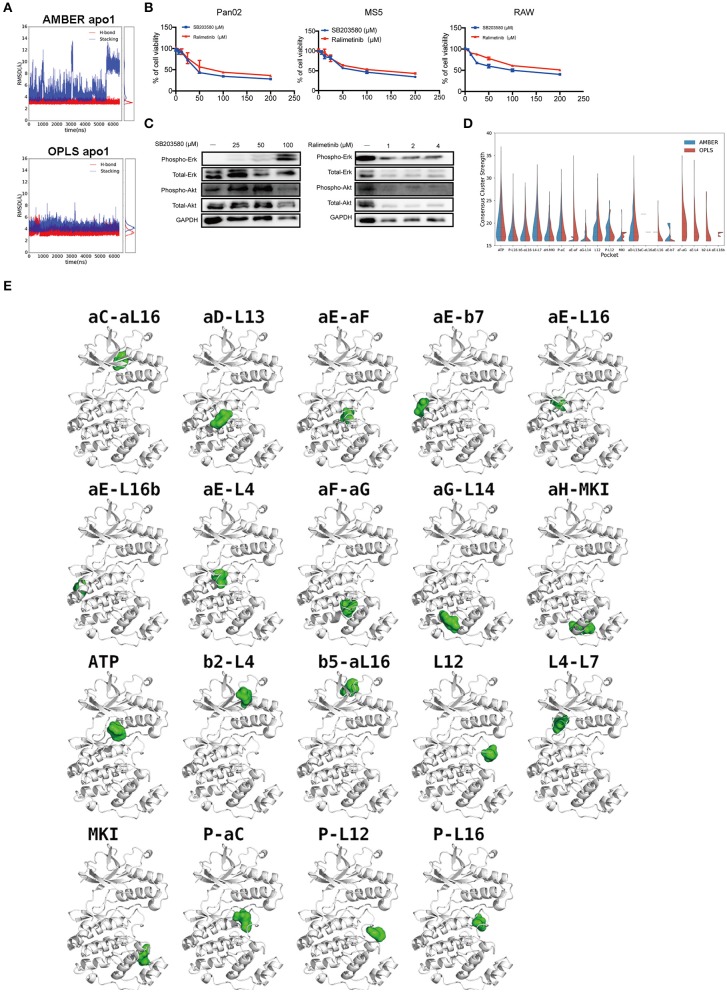
p38α-SB203580 interaction and potential binding pockets in p38α. **(A)** Distance plots of hydrogen bond and stacking interactions between p38α and the inhibitor SB203580. The hydrogen bond distance is measured between backbone amide of MET109 and atom NB1 in the inhibitor (red lines). The stacking interaction distance is measured between the center of mass of the six-member ring in TYR35 and the inhibitor (blue lines). Probability density functions are shown in the side panel. **(B)** Cell viabilities of Pan02, RAW, MS5 cell lines treated with 3.125–50 μM SB203580 for 24 h (*n* = 6 samples per group). Cell viabilities of Pan02, RAW, MS5 cell lines treated with 1.25–10 μM ralimetinib for 24 h (*n* = 6 samples per group). **(C)** SB203580 and ralimetinib inhibit phosphorylation of ERK and AKT at 4 h and 30 min in Pan02 cells. GAPDH indicates the loading level in each lane. (*n* = 3 samples per group) **(D)** Violin plot of consensus cluster strength for potential ligand-binding pockets identified from AMBER (blue) and OPLS (red) simulations of apo p38α. Results with consensus cluster strength *S* ≥ 16 are plotted with quartiles shown as dashed lines inside the violins. Note that for pockets with very few occurrences in simulations, only single lines are displayed. **(E)** Representative snapshots showing the location of potential ligand-binding pockets identified in FTMap analysis of AMBER and OPLS simulations. p38α protein is shown in white ribbon representation and pockets are indicated by corresponding solvent probes shown in green surface representation. Note that the apo p38α crystal structure (PDB code: 1P38) is used here for consistency in visualization.

The commonly used p38α inhibitor, SB203580, was suggested as a potential drug for cancer therapy (37). To study the drug-kinase dynamics of p38α, we measured the distances of two major interactions: (1) the hydrogen bond between the backbone of MET109 and SB203580; (2) the π-π stacking interactions between the phenyl sidechain of TYR35 and SB203580. In AMBER simulations, the hydrogen bond is well-maintained; the π-π stacking interaction is frequently disrupted with ring-ring distance up to 13 Å (Figure 6A). In OPLS simulations, although with minor fluctuations, both the hydrogen bond and the π-π stacking interaction are well-maintained (Figure 6A). SB203580 is less stable in AMBER simulations than in OPLS simulations, which is consistent with the wider range of RMSD fluctuations in AMBER simulations (Figure 3A, blue lines), suggesting that the ligand stability might affect the protein flexibility and vice versa.

### Current p38α Inhibitor Is Not Highly Selective

Although a variety of p38α inhibitors have been developed with enhanced specificity (38), most of these inhibitors are ATP competitors. Due to the similarity of the ATP-binding site of different kinases, off-target effects remain one of the biggest obstacles for the clinical application of p38α inhibitors. We tested the p38α inhibitors in PDAC cancer cells and various host cell lines. Indeed, both first and second generation of p38α inhibitors impedes cell viability of healthy host fibroblasts, and monocytes in a similar pattern as of cancer cells (Figure 6B). Surprisingly, in PDAC cells treated with two classical inhibitors, the phosphorylation of other kinases such as AKT, ERK is significantly altered under a relatively modest concentration (Figure 6C). Interestingly, the total amount of AKT was reduced under 100 μM SB203580 and 1 μM ralimetinib treatment, suggesting that an indirect regulatory pathway might be involved (Figure 6C). These findings suggest current p38α inhibitor is not selective enough. New approaches to inhibit the p38α needs to be explored for PDAC treatment.

### Potential Drug Binding Sites in p38α

To explore potential ligand-binding pockets in p38α, computational solvent mapping was performed using FTMap (39, 40). Pockets with consensus cluster strength *S* ≥ 16 are considered druggable. Using these criteria, 19 druggable pockets were found (Table 5): αD-L13 pocket, L4-L7 pocket, P-αC pocket, αG-L14 pocket, αE-L16 pocket, αE-L16b pocket, P-L12 pocket, L12 pocket, αE-β7 pocket, αC-αL16 pocket, β5-αL16 pocket, P-L16 pocket, MKI pocket, ATP pocket, αE-αF pocket, αH-MKI pocket, αF-αG pocket, β2-L4 pocket, αE-L4 pocket (Figure 6D). These pockets have a varied frequency of occurrences in simulations (Table 6) and are scattered in the entire p38α structure (Figure 6E). For comparison, FTMap analysis performed on crystal structure ensemble consisting of 196 crystal structures of p38α identified 6 druggable pockets: P-L16 pocket, ATP pocket, L4–L7 pocket, αG-L14 pocket, β5-αL16 pocket, MKI pocket, which partially verified our simulation results. Besides the well-studied ATP pocket and MKI pocket, the remaining 17 pockets are novel pockets that may be explored for further cancer drug development. Interestingly, a comparison between apo and ligand-bound simulations indicates that the presence of the ligand seems to prevent the exposure of a few binding pockets in AMBER (Figure S14A) and OPLS simulations (Figure S14B). Taken together, we provide potential new drug-binding sites that may pave the way for new generation drug design targeting p38α.

**Table 5:** Details of pocket lining residues of potential ligand-binding pockets identified from FTMap analysis.

**Pocket name**	**Residue #**
aD-L13	118, 183, 221
L4-L7	81, 82, 83, 84, 86
P-aC	67, 74, 171
aG-L14	222, 237, 273
aE-L16	140, 317, 320
aE-L16b	125, 132, 311
P-L12	193, 197, 199
L12	177, 185, 194
aE-b7	116, 126, 162
aC-aL16	73, 76, 344
b5-aL16	88, 92, 346
P-L16	145, 146, 70, 325, 326
MKI	242, 249, 259
ATP	34, 35, 169, 109
aE-aF	142, 202, 299
aH-MKI	241, 269, 289
aF-aG	207, 214, 235
b2-L4	16, 17, 57
aE-L4	82, 134, 137

**Table 6:** Number of occurrences of potential ligand-binding pockets in MD simulations.

**Pocket name**	**AMBER apo**	**OPLS apo**	**AMBER bound**	**OPLS bound**
aD-L13	264	2061	20	30
L4-L7	3691	564	723	674
P-aC	129	1137	218	186
aG-L14	26	91	3	179
aE-L16	1	49	N/A	N/A
aE-L16b	N/A	3	N/A	N/A
P-L12	66	11	28	8
L12	70	539	47	43
aE-b7	3	43	2	45
aC-aL16	1	1	5	N/A
b5-aL16	359	466	32	67
P-L16	978	742	1121	662
MKI	14	4	1	N/A
ATP	9,553	4,371	3,738	2,498
aE-aF	3	4,641	2	63
aH-MKI	176	111	37	17
aF-aG	N/A	117	N/A	N/A
b2-L4	N/A	79	6	N/A
aE-L4	N/A	121	N/A	N/A

*Note that the total number of snapshots used for FTMap analysis are 13,114 (AMBER apo),13,462 (OPLS apo), 6,468 (AMBER bound), and 6,347 (OPLS bound), respectively*.

## Discussion

The role of p38 MAPKs in cancer is still under intense investigation. What is the role of p38 MAPK family members in cancer? The one-word answer is “context-dependent.” Some human tumors, such as HCCs, have lower p38 MAPK activity than non-tumorigenic tissues (41). Several reports showed that p38α is a tumor suppressor. In support of that view, negative regulators of p38α, such as the phosphatases PPM1D and DUSP26, are overexpressed in human tumors (42, 43). However, bearing the evidence of p38α signaling in tumor suppression, mutations of p38α have not been consistently identified in human tumors. It is suspected that cancer cells may benefit from the p38α signaling pathway. In support of that view, p38α blockades show significant tumor suppressing effects *in vivo* in various cancer types (44, 45), suggesting the dual roles of p38α signaling in cancer. Here in our work, a thorough screening for more than 30 types of tumors and their counterparts shows that *p38*α expression is significantly increased in PDAC tissues, which drives our curiosity. We originally hypothesized that p38α may also be activated in PDAC cells and p38α blockades may benefit PDAC patients. Our experimental data support this hypothesis, and SB203580 significantly inhibit human and mouse PDAC cell growth. Moreover, in our recently unpublished data, SB203580 reduces the activity of pancreatic tumor derived-macrophages in *in vivo* models. Our data suggest that p38α blockades may block various cell components in PDAC tumor microenvironment, and may serve as a potential target for PDAC therapy.

Clinically, obesity is associated with cancer risk (46), and the adipose tissue microenvironment supports cancer development, metastasis (47), and drug resistance (29). For tumors predominantly occur in the adipocyte-rich microenvironments such as breast, prostate, ovarian, colon, and pancreatic cancers, the degree of adipose tissue involvement is often correlated with poor prognosis (48). Our data shows that *MAPK14* is correlated with *PLIN2* and *PLIN3*, but not *PLIN1, 4*, and *5*. Notably, PLIN2 and 3 is the marker for small lipid droplets. These results may reflect that cancer cells induce lipolysis of surrounding adipocyte, which is previously reported in breast cancer (49).

After establishing the role of p38α in PDAC cells, we turn to computational biology tools for more detailed information on the atomistic level for this interesting target. We performed molecular dynamics study on p38α conformational dynamics on the state of the art, highly parallel supercomputer, Anton. Supercomputer-powered biomolecular simulation provides us abundant information related to p38α function. Initially, a powerful and direct metrics, all-too-all RMSDs were calculated to examine the convergence of MD simulations. We observed the frequent appearance of low RMSD stripes in both AMBER and OPLS simulations (Figure S3A, dark blue areas), indicating a reasonable convergence. The results show that across different simulation runs, the conformational similarity is quite low, especially for OPLS apo1 (6.3 μs), which is significantly different from all other simulation runs (Figure S3B, mid panel, lower panel, red areas), possibly due to large conformational change of the glycine-rich loop in OPLS apo1 simulation (Figure S5). We found 11 p38α crystal structures with glycine-rich loop conformations that at least partially resemble the unfolded conformation found in MD simulations, suggesting that the novel conformation of the glycine-rich loop might be realistic.

The high flexibility we found in local regions of apo p38α is generally in line with previous simulation studies. Kuzmanic et al., performed metadynamics simulations of p38α to find that the glycine-rich loop, the activation loop, and the L16 loop are highly flexible (50). McClendon et al. carried out multiple microsecond-scale MD simulations of protein kinase A (PKA) and observed the opening and closing motions of the glycine-rich loop (51). Shan et al. performed long MD simulations of EGFR kinase to find that it is intrinsically disordered in the glycine-rich loop and the activation loop (52). Kumar et al. performed long MD simulations of Aurora-A kinase and identified several highly flexible regions including the glycine-rich loop and the activation loop (53). These data support that the conformational flexibility of local regions might be a common feature in protein kinases, presumably conferring adaptability in enzyme catalysis and protein-protein interactions.

The “butterfly” motion identified in PCA analysis is a quite interesting protein dynamics motion. Similar motions were seen in simulations of other enzymes as well such as the adenylate kinase (54). Many two-lobe enzymes may show similar type of motions during catalysis. Meanwhile, it is possible that both “conformational selection” and “induced fit” (55–57) contribute to the ligand-binding because the PC1-PC2 subspace of apo and ligand-bound p38α has both overlapping and non-overlapping regions. Nevertheless, the PCA result is complicated by two factors. Firstly, the initial structure of apo and SB203580-bound p38α simulations are similar (with RMSD of only 0.5 Å). Therefore, it is highly likely to have structural overlaps. Secondly, the apo and SB203580-bound p38α crystal structures come from mouse and human, respectively. The two structures differ at residue 48 and 263 (HIS and ALA for the mouse form; LEU and THR for the human form), which may be a potential source of error.

Despite the different conformational changes, linear regression of simulated and experimental chemical shifts shows similarly good agreement in AMBER and OPLS simulations, indicating that chemical shift is not sensitive to conformational changes. In contrast, linear regression of simulated and experimental RDCs shows a lower correlation coefficient in OPLS apo1 than other simulation runs, suggesting that RDC is sensitive to conformational changes. Large conformational changes of both N-terminal and C-terminal domains in OPLS apo1 may also contribute to the relatively worse RDC agreement. Since accurate RDC predictions are dependent on the precise distribution of the surfaces charges (58), we calculated all-to-all distance matrix for all 85 charged residues (Table 4) in p38α including ARG, LYS, ASP, and GLU for the simulation run AMBER apo1, AMBER apo2, and OPLS apo1. The charged residue distance matrices have a much bigger discrepancy between AMBER apo1 and AMBER apo2 than between AMBER apo1 and OPLS apo1 (Figure S13), suggesting that surface charge distribution can be an important contributing factor in RDC predictions.

Newly discovered potential druggable pockets in this study may benefit millions of PDAC patients. Notably, ATP pocket, MAP kinase insert (MKI) pocket, and αC-αL16 pocket have been experimentally verified (13, 59). Several novel pockets are in proximity to the binding site of upstream activators or downstream substrates of p38α and can potentially be explored to modulate its activity. There are at least four pockets that can be potentially used for this purpose: (1) αD-L13 pocket, which can be used to block docking of kinase substrates; (2) αC-αL16 pocket, which can be used to lock p38α in inactivate conformation; (3) αG-L14 pocket, which can be used to block p38α-TAB1 interaction (60); (4) αE-β7 pocket, which can be used to block p38α-MK2 interaction (61) and p38α-MKP5 interaction (62). Interestingly, the binding of the inhibitor seems to veil many potential binding pockets in AMBER and OPLS simulations (Figure S14). Further, virtual screenings and experimental validations are needed to confirm the druggability of these novel pockets.

Taken together, our study provides an interesting potential target for combating PDAC and offers detailed conformational dynamics information on p38α protein. Our findings have also paved potential avenues for developing the new classes of p38α-targeting drug, which may overcome the side effects of current therapy and benefit PDAC patients.

## Materials and Methods

### Cell Culture

Murine Panc02 pancreatic ductal adenocarcinoma cell line was kindly provided by Dr. Maximilian Schnurr from Munich University, Germany. Murine monocyte/macrophage-like cell line RAW264.7 and human monocyte/macrophage-like cell line THP-1 were kindly provided by Dr. Dapeng Yan at the Fudan University, China. Human TERT-immortalized fibroblasts Whirly, human PDAC cell lines MiaPaCa-2, Murine PDAC cell Pan02, and Human umbilical vein endothelial cell HUVEC were kindly provided by Dr. Yihai Cao from the Karolinska Institutet, Sweden. Murine fibroblast cell line MS5 were purchased from the ATCC. Panc02, RAW264.7, MS5, Whirly, MiaPaCa-2 were cultured in 10% FBS-DMEM (Cat. No. TBD10569, TBD, China) containing 100 U/ml penicillin, 100 μg/ml streptomycin (Cat. No. MA0110, Meilunbio, China). THP-1 cells were cultured in 10 % FBS-RPMI1640 (Cat. No. HY1640, TBD, China) containing 100 U/ml penicillin, 100 μg/ml streptomycin. HUVEC cells were cultured in 10% FBS-Medium 199/EBSS (Cat. No. SH30253.01, HyClone) containing 100 U/ml penicillin, 100 μg/ml streptomycin. All cell lines used in our study were negative for mycoplasma (Cat. No. LT07-318; Lonsa).

### Human Patient Samples

All studies related to clinical human samples were approved by the Ethical Review Committee in Shuguang Hospital, Shanghai University of Traditional Chinese Medicine, Shanghai, China. Pancreatic tumor samples and adjacent pancreatic tissues were collected from cancer patients with written informed permission.

### Database Analysis

Transcriptome data from patient samples of pancreatic cancer were analyzed using the online database, The Cancer Genome Atlas (TCGA, https://www.cancer.gov/about-nci/organization/ccg/research/structural-genomics/tcga) to investigate whether the expression of interesting markers is altered in tumor tissue. RNA sequencing analysis and visualization platform Gene Expression Profiling Interactive Analysis (GEPIA, http://gepia.cancer-pku.cn/) (63) were used to perform the correlation analysis and survival analysis in pancreatic cancer cohort. A gene expression profile across various cancer types and paired normal samples was generated from GEPIA.

### Immunoblot

Fresh tumor tissues and cultured cells were lysed in RIPA buffer and the proteinase and phosphatase inhibitor cocktail (Cat. No. MA0151, Meilunbio, China; Cat. No. MB2678, Meilunbio, China; 1:100). For immunoblot, each protein sample and a standard molecular weight marker (Cat. No. WJ102, EpiZyme, China) were loaded onto a 10% SDS-PAGE gel (Cat. No. PG112, EpiZyme, China), followed by transferring onto a polyvinylidene difluoride (PVDF) membrane (Cat. No. IPVH00010, Millipore). The membranes were blocked with 5% skimmed milk for 2 h, and were probed overnight at 4°C with a rabbit anti-phospho-p38 antibody (Cat. No. 4631S, Cell Signaling; 1:1,000), a rabbit anti-p38 antibody (Cat. No. 9212S, Cell Signaling; 1:1,000), a rabbit anti-phospho-Erk1/2 antibody (Cat. No. 9101S, Cell Signaling; 1:1,000), a rabbit anti-Erk1/2 antibody (Cat. No. 4695S, Cell Signaling; 1:1,000), a rabbit anti-phospho-AKT antibody (Cat. No. 2118-1, Epitomics; 1:1,000), a rabbit anti-AKT antibody (Cat. No. 1085-1, Epitomics; 1:1,000) and a mouse anti-GAPDH antibody (Cat. No. A01020, Abbkine; 1:1,000) in 5% skimmed milk. After rigorous washing with PBS containing 0.1% Tween-20 (Cat. No. T8220, Solarbio, China), membranes were incubated with a goat anti-mouse HRP-conjugated IgG antibody (Cat. No. AS003, ABclonal; 1:5,000) and a goat anti-rabbit HRP-conjugated IgG antibody (Cat. No. AS014, ABclonal; 1:5,000). Target proteins were visualized using a super-sensitive ECL luminescence reagent (Cat. No. MA0186, Meilunbio, China) with a Molecular Imager ChemiDoc XRS System (Bio-Rad).

### RNA Extraction and Quantitative Real-Time PCR

Total RNAs were extracted from tumor tissues and cultured cells using RNAsimple Total RNA kit (Cat. No. DP419, TIANGEN, China). Total RNA from each sample was reversely transcribed using an All-in-One cDNA Synthesis SuperMix (Cat. No. B24408, Bimake, China). Reverse transcription was performed at 42°C for 60 min, followed by 70°C for 5 min to inactivate the enzyme activity. cDNA samples were stored at −20°C and subjected to qPCR using a StepOnePlus Real-Time PCR System (Applied Biosystems). Each qPCR sample was performed in a triplicate and 10 μl reaction containing 2 × SYBR Green qPCR Master Mix (Cat. No. B21202, Bimake, China), 50 nM forward and reverse primers, and 4 μl cDNA. The qPCR protocol was executed for 45 cycles and each cycle consisted of denaturation at 95°C for 15 s, annealing at 60°C for 1 min, and extension at 72°C for 1 min. The primer pairs used in our experiments included: mouse *Gapdh* forward: 5′-CCAGCAAGGACACTGAGCAA-3′; mouse *Gapdh* reverse: 5′-GGGATGGAAATTGTGAGGGA-3′; mouse *Mapk14* forward: 5′- GGGACACCCCCTGCTTATCT-3′; mouse *Mapk14* reverse: 5′- TCCCTGCTTTCAAAGGACTGG-3′; mouse *Mapk11* forward: 5′- GGACCTGAACAGGATCGTGAA-3′; mouse *Mapk11* reverse: 5′- CTCAGCCATGAAGCCTCCC-3′; mouse *Mapk12* forward: 5′- CGCCGTGTACCAAGACCTG-3′; mouse *Mapk12* reverse: 5′- GAGGCGCAACTCTCTGTAGG-3′; mouse *Mapk13* forward: 5′- GAGGCGCAACTCTCTGTAGG-3′; mouse *Mapk13* reverse: 5′- CACTCAGGGTCTCATGCTTCA-3′; human *GAPDH* forward: 5′-AGGGCTGCTTTTAACTCTGGT-3′; human *GAPDH* reverse: 5′-CCCCACTTGATTTTGGAGGGA-3′; human *MAPK14* forward: 5′- CCCGAGCGTTACCAGAACC-3′; human *MAPK14* reverse: 5′- TCGCATGAATGATGGACTGAAAT-3′; human *MAPK11* forward: 5′-AAGCACGAGAACGTCATCGG-3′; human *MAPK11* reverse: 5′- TCACCAAGTACACTTCGCTGA-3′; human *MAPK12* forward: 5′- CCCAGACATCAGGGAGTAATGG-3′; human *MAPK12* reverse: 5′- TCTATCGGATACTTCAGCGTCA-3′; human *MAPK13* forward: 5′- CACTCAGGGTCTCATGCTTCA-3′; human *MAPK13* reverse: 5′- GCTTGCGTTGGTCAGGACA-3′.

### Histology and Immunohistochemistry

Paraffin-embedded tissues were cut in the thickness of 5 μm, mounted onto glass slides, baked for 1 h at 60°C, deparaffinized in Xylene (Cat. No. 10023418, SCR, China), and sequentially rehydrated in 99, 95, and 70% ethanol (Cat. No. 10009218, SCR, China). Tissue slides were counterstained with Haematoxylin (Mayer's) (Cat. No. MB9897, Meilunbio, China) and Eosin (Cat. No. MA0164, Meilunbio, China) before dehydration with 95 and 99% ethanol, and were mounted with neutral balsam (Cat. No. 1004160, SCR, China). Stained tissues were analyzed under a light microscope (Leica DM IL LED). For immunohistochemical staining, paraffin-embedded tissue sections were stained with a rabbit anti-PDGFRβ antibody (Cat. No. ab32570, Abcam; 1:200); a mouse anti-CD163 antibody (Cat. No. ab156769, Abcam; 1:200); a rabbit anti-αSMA antibody (Cat. No. ab32575, Abcam; 1:200); a rabbit anti-phospho-p38 antibody (Cat. No. 4631S, Cell Signaling; 1:100), and a rabbit anti-p38 antibody (Cat. No. 9212S, Cell Signaling; 1:100). A ready to use HP IHC detection kit (Cat. No. abs957, Absin, China) was used for visualization. Captured images were further analyzed using Adobe Photoshop CS software.

### Cell Viability

Panc02, RAW, and MS5 cells at the density of 1 × 10^4^ cells per well were seeded in a 96-well plate. After 24 h of incubation, cells were treated with 3.125–50 μM SB203580 SB203580 (Cat.No.S1076, selleck,USA) and 1.25–10 μM ralimetinib (Cat. No.S1494, selleck,USA). Cell viability was measured using a Cell Counting Kit-8 (Cat. No. MA0218-5, Meilunbio, China). In brief, 10 μl of the solution containing 2-(2-methoxy-4-nitrophenyl)-3-(4-nitrophenyl)-5-(2,4-disulfophenyl)-2H-tetrazolium (WST-8) was added to each well of a 96-well plate, followed by incubation for 4 h. Densitometry was measured at the wavelength of 450 nm by a Synergy 2 Multi-Mode Microplate Reader (BioTek).

### Simulation System Preparation

p38α protein structures were prepared using Maestro 9.0 (Schrödinger, LLC). The crystal structures of apo p38α (PDB code: 1P38) (9) and SB203580-bound (PDB code: 1A9U) p38α (10) were used as initial structures. The protein was capped with acetyl group (ACE) at the N-terminus and N-methyl group (NME) at the C-terminus to prevent artificial charge-charge interactions. AMBERff99SB-ILDN (64) and OPLS-AA/L (65) force fields were used for the protein. A box size of 85.0 × 85.0 × 85.0 Å^3^ was used to ensure the enclosure of the entire protein. Apo p38α or SB203580-bound p38α system was solvated in water described by the TIP3P model (66) to mimic physiological condition and neutralized using nine sodium ions (67).

### Ligand Parameterization

To derive parameters for the SB203580, we used different approaches. For AMBER simulation, the bonded parameters and non-bonded parameters for SB203580 were parameterized using General Amber Force Field (GAFF) (68). The partial charges were derived using the restrained electrostatic potential (RESP) method on the RED server (69, 70). For OPLS simulations, the bonded parameters, non-bonded parameters, and partial charges were derived by analogy method as previously described (71). In both simulations of ligand-bound p38α, dihedral restraints were applied to eight dihedral angles to keep the ligand conformations close to the crystal structures. The force constant for dihedral restraints is 4184 kJ/mol.rad. Each equilibrium angle (Table 1) was obtained from four SB203580-bound p38α crystal structures [PDB codes: 1A9U (10), 1PME (72), 2EWA (17), and 3GCP (73)].

### Molecular Dynamics Simulations

Before the production simulation, energy minimization, and equilibration were performed on our in-house clusters using Desmond 2.4 (74). Energy minimization was initially carried out using the steepest descent algorithm for 1,000 steps and the L-BFGS algorithm for 1,000 steps. The energy-minimized structure was incrementally heated up from 50 to 298 K (50, 100, 150, 200, 250, 298 K) with 100 ps at each temperature in the NVT ensemble using Berendsen thermostat (75). Next, the protein was equilibrated at 298 K for an additional 10 ns in the NVT ensemble using the Nosé-Hoover thermostat (76, 77). For van der Waals interactions and short-range electrostatic interactions, a 9 Å cutoff was used; for long-range electrostatic interactions, Particle Mesh Ewald (PME) was used (78). The “bonded,” “near,” and “far” time steps are 1, 1, and 3 fs, respectively.

### Anton Supercomputer Molecular Dynamics Simulations

For production simulations, we performed simulations on the Anton supercomputer (Pittsburgh supercomputing center, Pittsburgh, PA) (33). Production simulations were performed in the NVT ensemble with the temperature maintained at 298 K using Nosé-Hoover thermostat (76, 77). For van der Waals interactions and short-range electrostatic interactions, the cutoff was automatically determined by the “guess_chem” utility on Anton for optimized performance. Gaussian Split Ewald (GSE) (79) method was used for long-range electrostatic interactions. M-SHAKE algorithm (80) was used to constrain all bonds, enabling a 2 fs time step. Snapshots of the simulations were saved at an interval of 0.1 ns for analysis. Visualizations of simulation snapshots and trajectories were done using both PyMOL 1.8 (PyMOL 2015) and VMD 1.9 (81). For AMBER and OPLS simulations, three independent simulations of apo p38α and one simulation of SB203580-bound p38α were performed with detailed parameters shown in Table 2.

### Calculations of RMSD and RMSF

To obtain root mean square deviations (RMSDs) in p38α simulations, the trajectory (non-terminal residues, residue 14–344) at an interval of 0.1 ns was fitted to the initial structure on protein backbone; RMSDs were calculated for each snapshot over simulation time. To obtain all-to-all RMSDs, the trajectory at an interval of 1 ns was fitted to the initial structure on the protein backbone before all-to-all RMSD matrix was generated using pytraj (82), a Python package binding to cpptraj program (83). To obtain root mean square fluctuations (RMSFs) in p38α simulations, the trajectory at an interval of 0.1 ns was fitted to the initial structure on protein backbone; RMSFs were then calculated for each residue.

### Principal Component Analysis

Principal components analysis (PCA) was performed for a combined trajectory of apo and ligand-bound p38α for each force field. The input trajectory was initially aligned on Cα atoms of p38α and used to extract eigenvectors and eigenvalues. Then, the trajectory of Cα atoms was projected onto the two most dominant eigenvectors to obtain principal components PC1 and PC2. For experimental comparison, PCA of 44 full-length crystal structures of p38α was performed with PDB entries as follows: 5LAR, 5ETI, 5ETC, 4LOO, 3U8W, 3S3I, 3RIN, 3PY3, 3NNW, 3NNV, 3NNU, 3KQ7, 3ITZ, 3GFE, 3GC7, 3DT1, 3D83, 3D7Z, 2ZB0, 2YIX, 2I0H, 1ZZL, 1YQJ, 1WBW, 1WBV, 1WBT, 1WBS, 1WBO, 1WBN, 1W84, 1W83, 1W82, 1W7H, 1R3C, 1P38, 1OVE, 1OUY, 1OUK, 1M7Q, 1DI9, 1BMK, 1BL7, 1BL6, 1A9U. All PCA calculations were performed using the analysis tools g_covar and g_anaeig in Gromacs 5.0 (84, 85).

### Calculations of Chemical Shifts and Residual Dipolar Couplings

To obtain chemical shifts of the backbone atoms in p38α from MD simulations, we performed ensemble-based chemical shift calculations on MD trajectory at an interval of 1 ns using the PPM program (86). PPM predicts the chemical shifts of CA, CB, C, N, and H based solely on the physical and chemical properties of the protein structure. For comparison, we also performed chemical shift calculations on MD trajectory at an interval of 1 ns using the SHIFTX2 program (87). SHIFTX2 predicts the chemical shifts of CA, CB, C, N, and H using both structure- and sequence- based criteria. Experimental p38α chemical shifts were obtained from Biological Magnetic Resonance data bank (BMRB entry number: 6468) (88); the entry includes 219 ^1^H chemical shifts, 684 ^13^C (including CA, CB, and C) chemical shifts, and 219 ^15^N chemical shifts for apo p38α. For comparison of simulated and experimental chemical shifts of atom CA, CB, C, N, and H, different residues were used depending on the availability of experimental chemical shifts for specific atoms in BMRB entry 6,468. To obtain residual dipolar couplings (RDCs) of each residue in p38α, we performed RDC calculations on structural snapshots at an interval of 1 ns using the PALES program (58, 89). Experimental RDC data for 39 residues of apo p38α and the parameters in RDC (Table 3) were obtained from previous report (36). Specifically, a concentration of bacteriophage Pf1 at 20 mg/mL and a pH of 6.0 was used. The sodium chloride concentration was optimized using a range from 0.005 to 0.5 M and 0.2 M was used for final RDC calculations.

### FTMap Analysis

A standalone version of FTMap (39, 40) was used to perform calculations on MD snapshots at an interval of 1 ns. FTMap analysis mainly consists of three steps: (1) independent docking of 16 organic probes on the protein using a fast Fourier transform correlation approach; (2) refinement of the probe positions using energy minimizations; (3) clustering and ranking of the resulting poses to identify consensus sites (CSs). The 16 solvent probes used in FTMap algorithm are: acetamide, acetonitrile, acetone, acetaldehyde, methylamine, benzaldehyde, benzene, isobutanol, cyclohexane, N, N-dimethylformamide, dimethyl ether, ethanol, ethane, phenol, isopropanol, urea. Consensus cluster strength (S) is defined as the number of probe clusters in a specific binding pocket on the protein. For experimental comparison, 196 crystal structures were also used for FTMap analysis. Their PDB codes are: 1A9U, 1BL6, 1BL7, 1BMK, 1DI9, 1IAN, 1KV1, 1KV2, 1LEW, 1LEZ, 1M7Q, 1OUK, 1OUY, 1OVE, 1OZ1, 1P38, 1R39, 1R3C, 1W7H, 1W82, 1W83, 1W84, 1WBN, 1WBO, 1WBS, 1WBT, 1WBV, 1WBW, 1WFC, 1YQJ, 1YW2, 1YWR, 1ZYJ, 1ZZ2, 1ZZL, 2BAK, 2FSL, 2FSM, 2FSO, 2FST, 2GFS, 2GHL, 2GHM, 2GTM, 2GTN, 2I0H, 2NPQ, 2PUU, 2QD9, 2RG5, 2RG6, 2Y8O, 2YIS, 2YIW, 2YIX, 2ZAZ, 2ZB0, 2ZB1, 3BV2, 3BV3, 3BX5, 3CTQ, 3D7Z, 3DS6, 3DT1, 3FC1, 3FI4, 3FKL, 3FKN, 3FKO, 3FLN, 3FLQ, 3FLS, 3FLW, 3FLY, 3FLZ, 3FMH, 3FMJ, 3FMK, 3FML, 3FMM, 3FMN, 3FSF, 3FSK, 3GC7, 3GCP, 3GCQ, 3GCS, 3GCV, 3GFE, 3GI3, 3HA8, 3HEC, 3HEG, 3HL7, 3HLL, 3HP2, 3HP5, 3HRB, 3HUB, 3HUC, 3HV3, 3HV4, 3HV5, 3HV6, 3HV7, 3HVC, 3ITZ, 3IW5, 3IW6, 3IW7, 3IW8, 3K3I, 3K3J, 3KF7, 3KQ7, 3L8S, 3LFA, 3LFB, 3LFC, 3LFE, 3LFF, 3MGY, 3MH0, 3MH1, 3MH2, 3MH3, 3MPA, 3MPT, 3MVL, 3MVM, 3MW1, 3NEW, 3NNU, 3NNV, 3NNW, 3NNX, 3NWW, 3O8P, 3O8T, 3O8U, 3OBG, 3OBJ, 3OC1, 3OCG, 3OD6, 3ODZ, 3P4K, 3P5K, 3P78, 3P79, 3P7A, 3P7B, 3P7C, 3PG3, 3PY3, 3QUD, 3QUE, 3RIN, 3ROC, 3S3I, 3U8W, 3UVP, 3UVQ, 3ZS5, 3ZSG, 3ZSH, 3ZYA, 4A9Y, 4AA0, 4AA4, 4AAC, 4DLI, 4DLJ, 4E5A, 4E5B, 4E6A, 4E6C, 4E8A, 4EH2, 4EH3, 4EH4, 4EH5, 4EH6, 4EH7, 4EH8, 4EH9, 4EHV, 4EWQ, 4F9W, 4F9Y, 4FA2, 4GEO, 4KIN, 4KIP, 4KIQ.

### Classification of Binding Pockets

Binding pockets are named and classified based on its adjacent secondary structures, which harbor pocket lining residues (Table 5). To determine the identity of a pocket, the intersecting volume of the bounding sphere for the probe clusters and the pocket lining residues were calculated for each pocket using the equation as described previously (90):

(1) $$V_{o}=\{\begin{array}{ccc} 0 & for & d\geq r_{1}+r_{2}\\ \frac{\pi }{12d}{\left(r_{1}+r_{2}-d\right)}^{2}\left(d^{2}+2d\left(r_{1}+r_{2}\right)-3{\left(r_{1}-r_{2}\right)}^{2}\right) & for & \left|r_{1}-r_{2}\right|<d<r_{1}+r_{2}\text{​​​}\\ \frac{4}{3}\pi {\left(\min\left\{r_{1},r_{2}\right\}\right)}^{3} & for & 0\leq d\leq \left|r_{1}-r_{2}\right| \end{array}$$

*V*_*o*_ is the intersecting volume; *r*_1_, *r*_2_ are the radii of the bounding sphere of the probe cluster and of the pocket lining residues, respectively; *d* is the distance between the two sphere centers. A specific probe cluster falls in a pocket category if it has the maximum intersecting volume with that pocket. A scaling factor of 0.75 on *r*_2_ was used to avoid the overestimation of the bound site volume. All pockets and subpockets in the ATP-binding site are classified as ATP pocket.

### Statistical Analysis

Statistical analyses of biological experiments were performed using the standard two-tailed Student *t*-test, and *p* < 0.05 was considered statistically significant.

## Data Availability Statement

The datasets analyzed in this study can be found in The Cancer Genome Atlas (https://portal.gdc.cancer.gov/).

## Ethics Statement

The studies involving human participants were reviewed and approved by Ethics committee, Shuguang Hospital, Shanghai University of Traditional Chinese Medicine. The patients/participants provided their written informed consent to participate in this study.

## Author Contributions

SZ generated the ideas, designed experiments, and wrote the manuscript. LY, XS, YY, and SZ performed most experiments and organized all figures. YL, JZ, WL, and AE participated in discussions.

### Conflict of Interest

The authors declare that the research was conducted in the absence of any commercial or financial relationships that could be construed as a potential conflict of interest.
