# Supplementary material for: p38α Mitogen-Activated Protein Kinase Is a Druggable Target in Pancreatic Adenocarcinoma
Source: Front Oncol. 2019 Nov 26;9:1294. doi: 10.3389/fonc.2019.01294 (PMC6890821; doi:10.3389/fonc.2019.01294)
Supplement: Supplementary file 1 [file Table_1.DOCX]

**Supplemental Figures and Figure Legends**

**p38α mitogen-activated protein kinase is a druggable target in pancreatic adenocarcinoma**

Ling Yang^1#^, Xiaoting Sun^2#^,Ying Ye^3#^ Yongtian Lu^4^, Ji Zuo^1^,Wen Liu^1^, Adrian Elcock^5^ and Shun Zhu^1*^

^1^Department of Cellular and Genetic Medicine, School of Basic Medical Sciences, Fudan University, Shanghai 200032, P.R. China

^2^Department of Medical Oncology, Shuguang Hospital, Shanghai University of Traditional Chinese Medicine, Shanghai, P.R. China

^3^Department of Oral Implantology, School and Hospital of Stomatology, Tongji University; Shanghai Engineering Research Center of Tooth Restoration and Regeneration, Shanghai, China.

^4^Department of ENT, Second People's Hospital of Shenzhen, First Affiliated Hospital of Shenzhen University, Shenzhen 518035, China.

^5^Department of Biochemistry, University of Iowa, Iowa City, Iowa 52242, United States

**Key words:** p38α, molecular dynamics, tumor targeted therapy, conformational dynamics, pancreatic cancer

^#^These authors contribute equally.

*Corresponding authors. Galley proofs and reprint requests should be addressed to: Shun Zhu, Ph.D., Department of Cellular and Genetic Medicine, School of Basic Medical Sciences, Fudan University, Shanghai 200032, P.R. China. Tel: (+86)- 13918136612, E-mail: shun_zhu@fudan.edu.cn


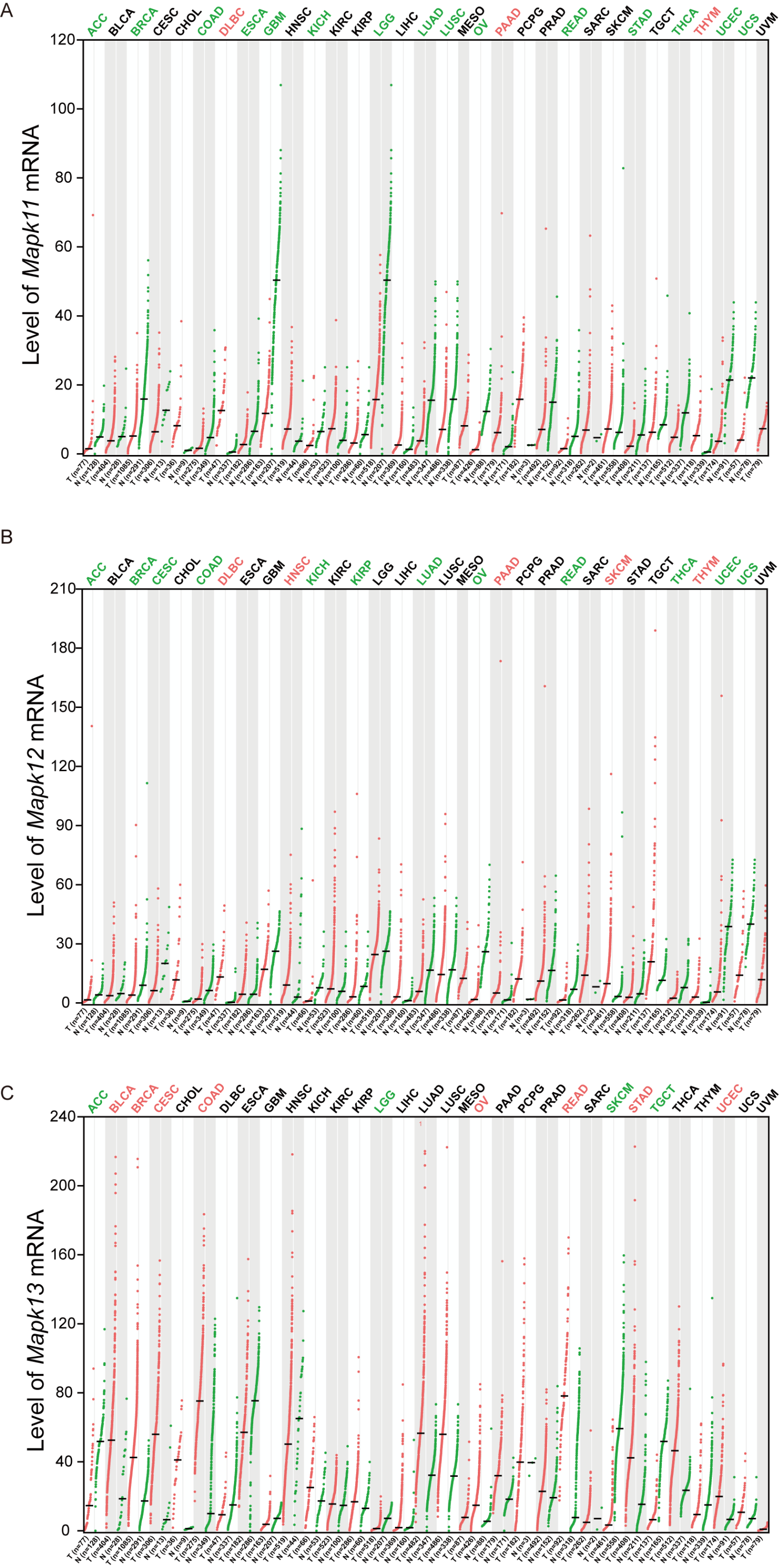


**Figure S1. Transcriptomic expression levels of p38 MAPK family members across multiple cancer types**

1. Transcriptomic expression levels of *MAPK11* across multiple cancer types and paired normal samples, with each dot representing a distinct tumor or normal sample. (B) Transcriptomic expression levels of *MAPK12* across multiple cancer types and paired normal samples, with each dot representing a distinct tumor or normal sample. (C) Transcriptomic expression levels of *MAPK13* across multiple cancer types and paired normal samples, with each dot representing a distinct tumor or normal sample. Red dot = tumor sample; Green dot = control sample; Red group name = significantly upregulated; Green group name = significantly downregulated; Black group name = not significant.


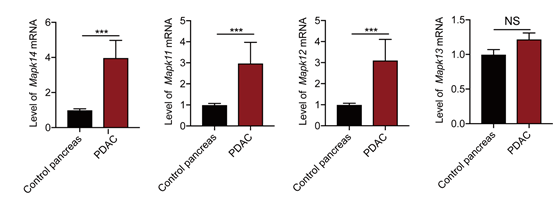


**Figure S2. Expression levels of p38 MAPK family members in human PDAC samples**

RNA expression levels of *MAPK14*, *MAPK11*, *­MAPK12* and *MAPK13* in human PDAC tissues and adjacent pancreas (n=20 samples per group). ***p<0.001. NS = not significant. Data presented as mean ± s.e.m..


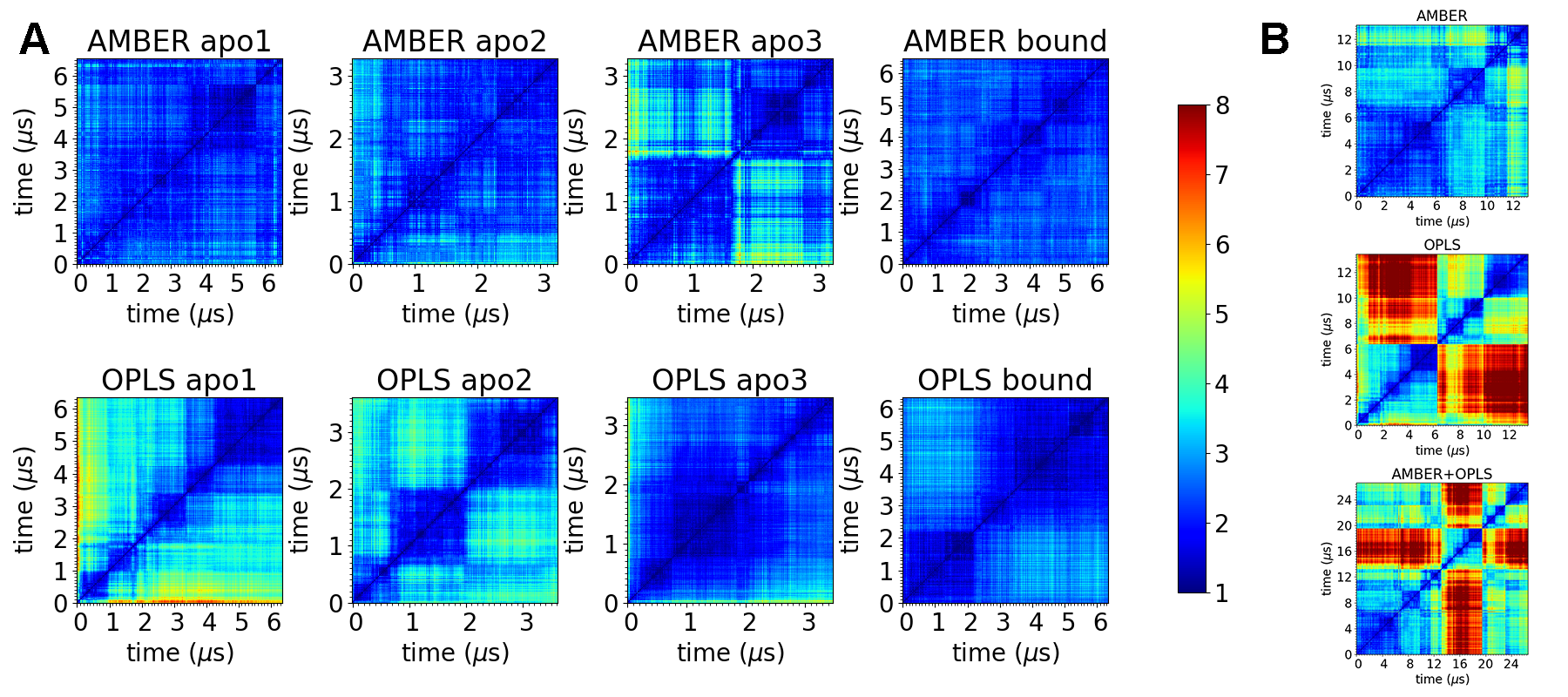


**Figure S3. All-to-all RMSD plots for p38α simulations.** (A) for AMBER and OPLS simulations individually. Backbone of the non-terminal residues (residues 14 to 344) was used for structural alignment and RMSD calculations of MD snapshots at an interval of 1ns. (B) for combined AMBER and/or OPLS simulations. Upper panel, combined runs apo1, apo2, and apo3 of AMBER simulations. Middle panel, combined runs apo1, apo2, and apo3 of OPLS simulations. Lower panel, combined trajectory of all simulation runs apo1, apo2, and apo3 in AMBER and OPLS simulations (the order is: AMBER apo1, AMBER apo2, AMBER apo3, OPLS apo1, OPLS apo2, and OPLS apo3).


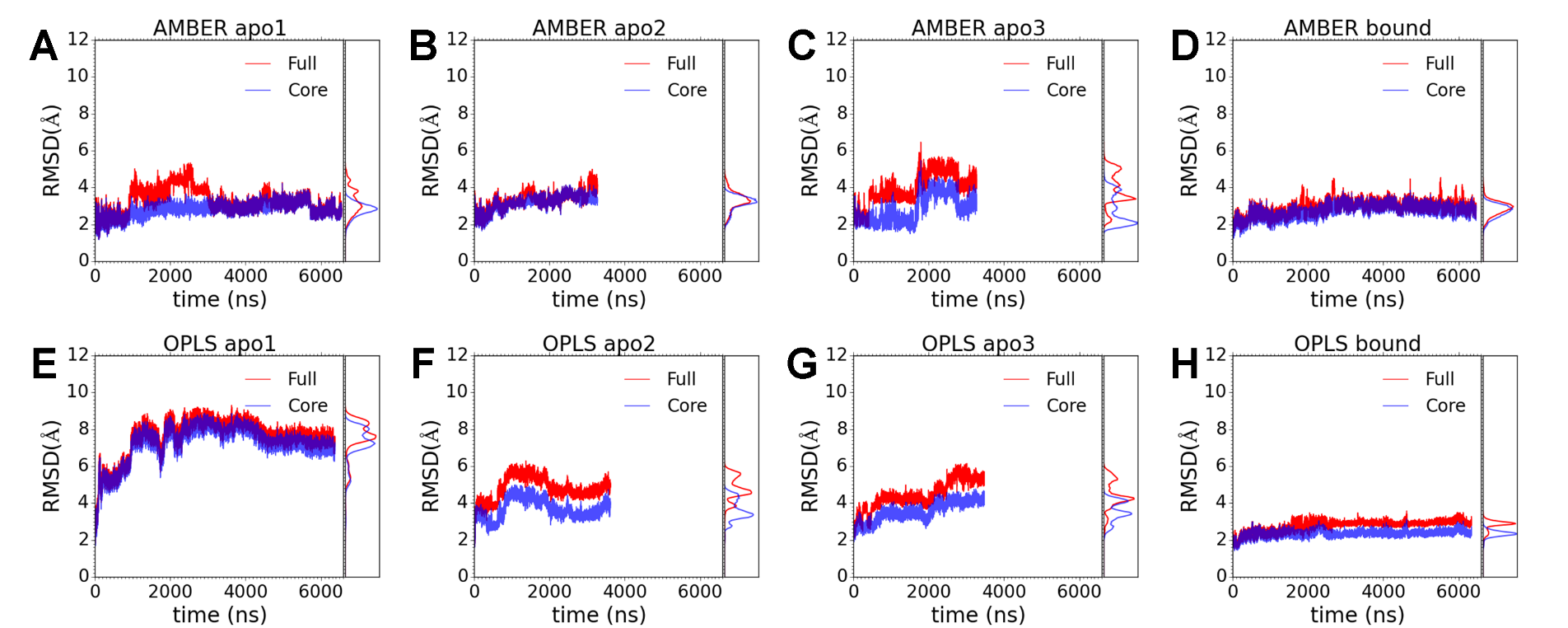


**Figure S4. RMSDs of p38α in solution simulations calculated with (Full) and without (Core) the terminals.** Results are shown for AMBER simulations (A)(B)(C)(D) and OPLS (E)(F)(G)(H) simulations. The terminals are defined to be the first and last 10 residues in p38α crystal structure, i.e., residues 4-14 and 345-354 (the numbering is consistent with p38α nomenclature).


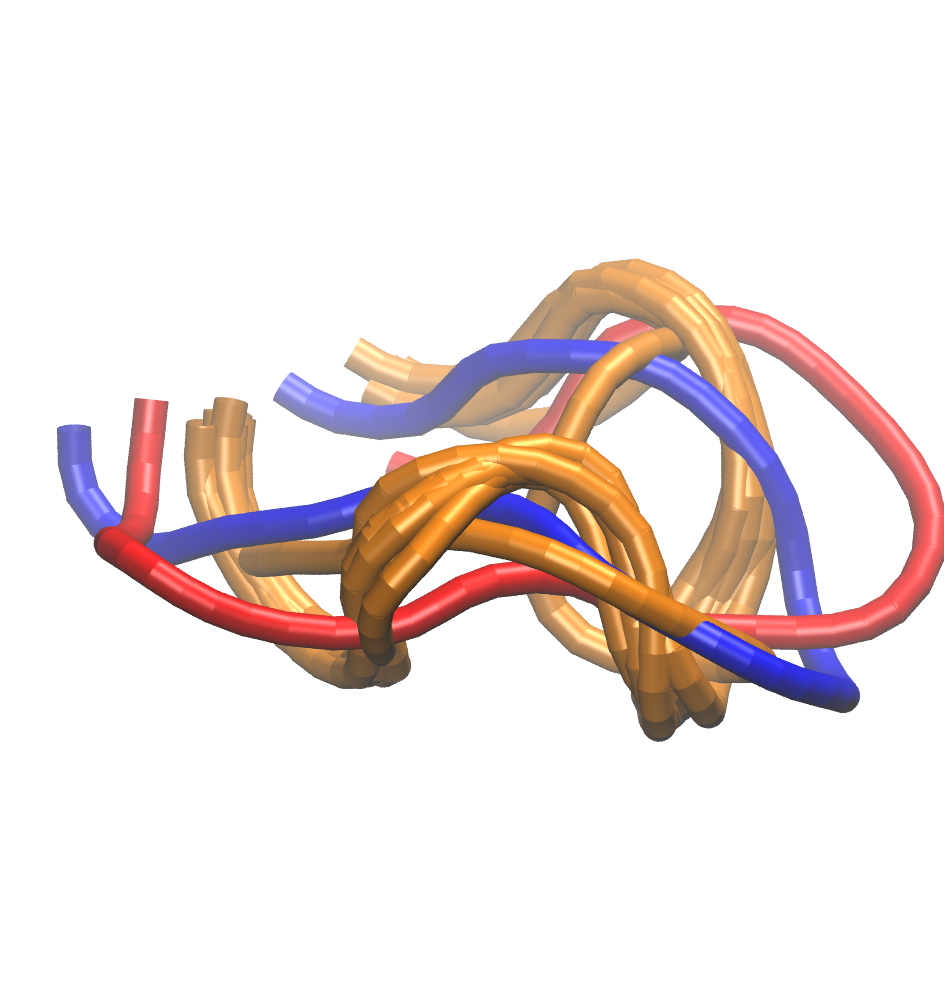


**Figure S5. Comparison of conformations of the glycine rich loop.** Blue and red ribbons show the glycine-rich loop at 0 ns and 6112.3 ns of OPLS apo1 simulation. Orange ribbons show the overlay of 11 structures that have unusual glycine-rich loop conformations (PDB codes: 1W7H, 1WBW, 1YQJ, 1ZZL, 2YIX, 3ITZ, 3RIN, 1OVE, 2I0H, 5LAR, 3D7Z).


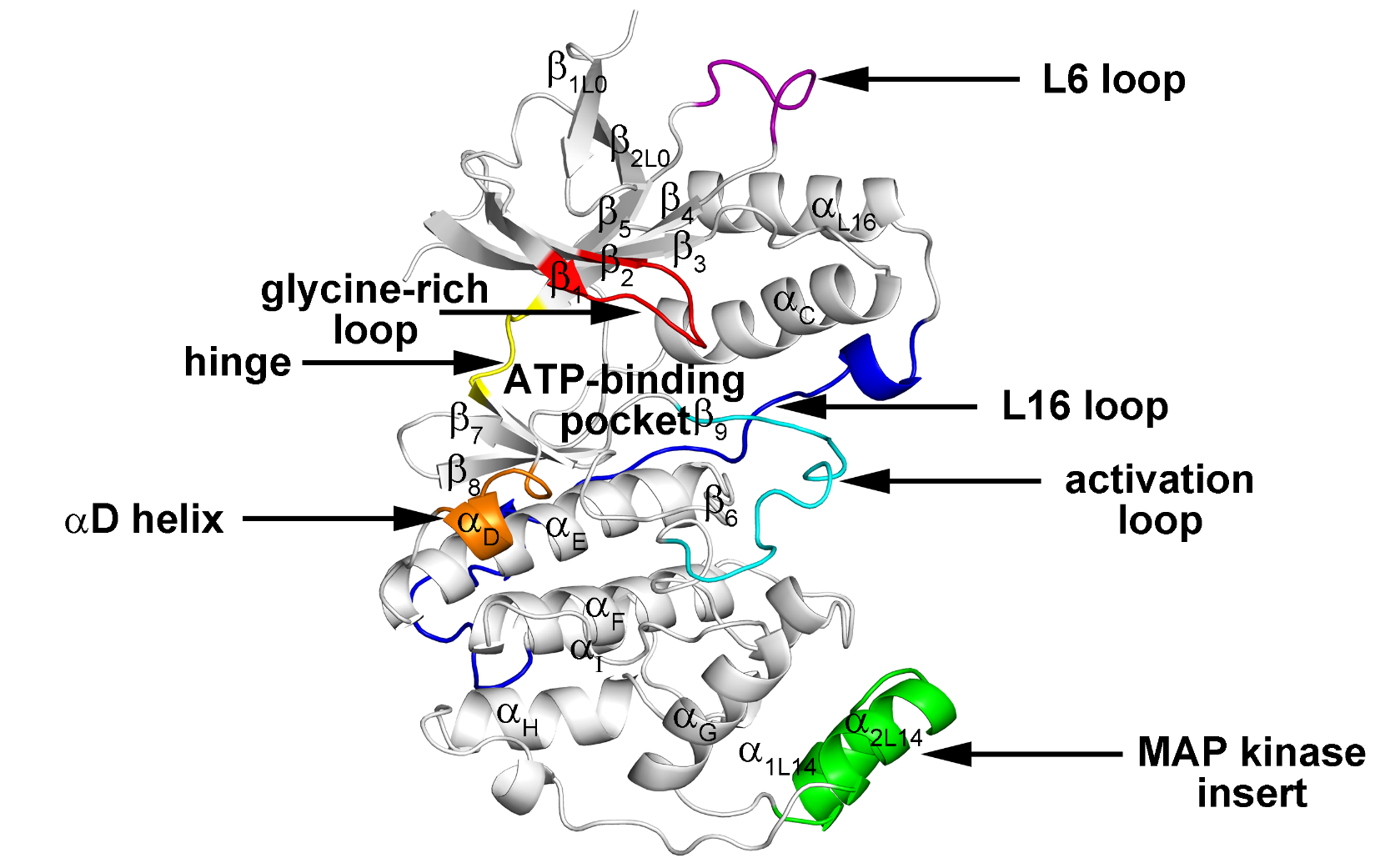

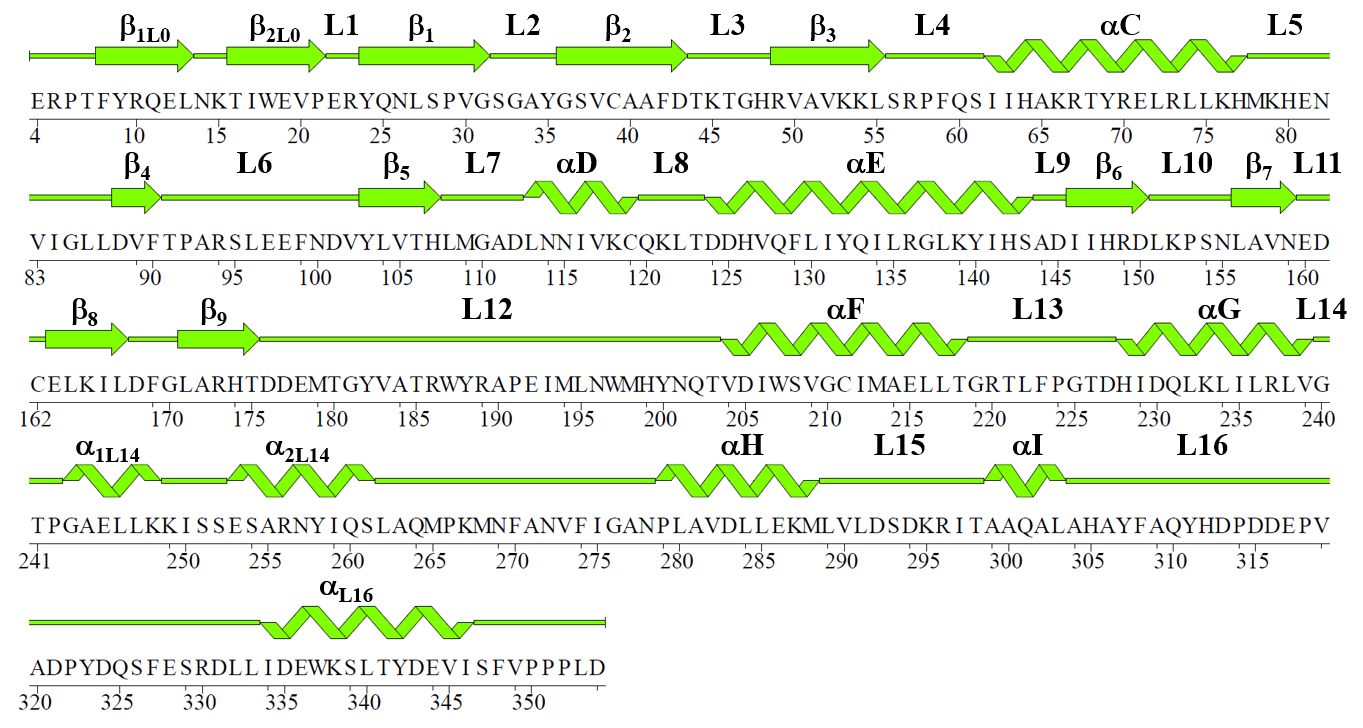


**A**

**B**

**Figure S6. Annotations of p38α structure and sequence.** (A) The structure of *apo* p38α are shown in ribbon representation with import local regions highlighted. They are the glycine-rich loop (red, residues 30 to 38), the L6 loop (purple, residues 93 to 99), the hinge (yellow, residues 107 to 110), the αD helix (orange, residues 113 to 119), the activation loop (cyan, residues 169 to 183), the MAP kinase insert (green, residues 243 to 261), and the L16 loop (blue, residues 305 to 330). (B) Wire plot showing the sequence and secondary structure in p38α (Wang et al. 1997) made with a modified version of PROCHECK v.3.5.4 (Laskowski et al., 1993).


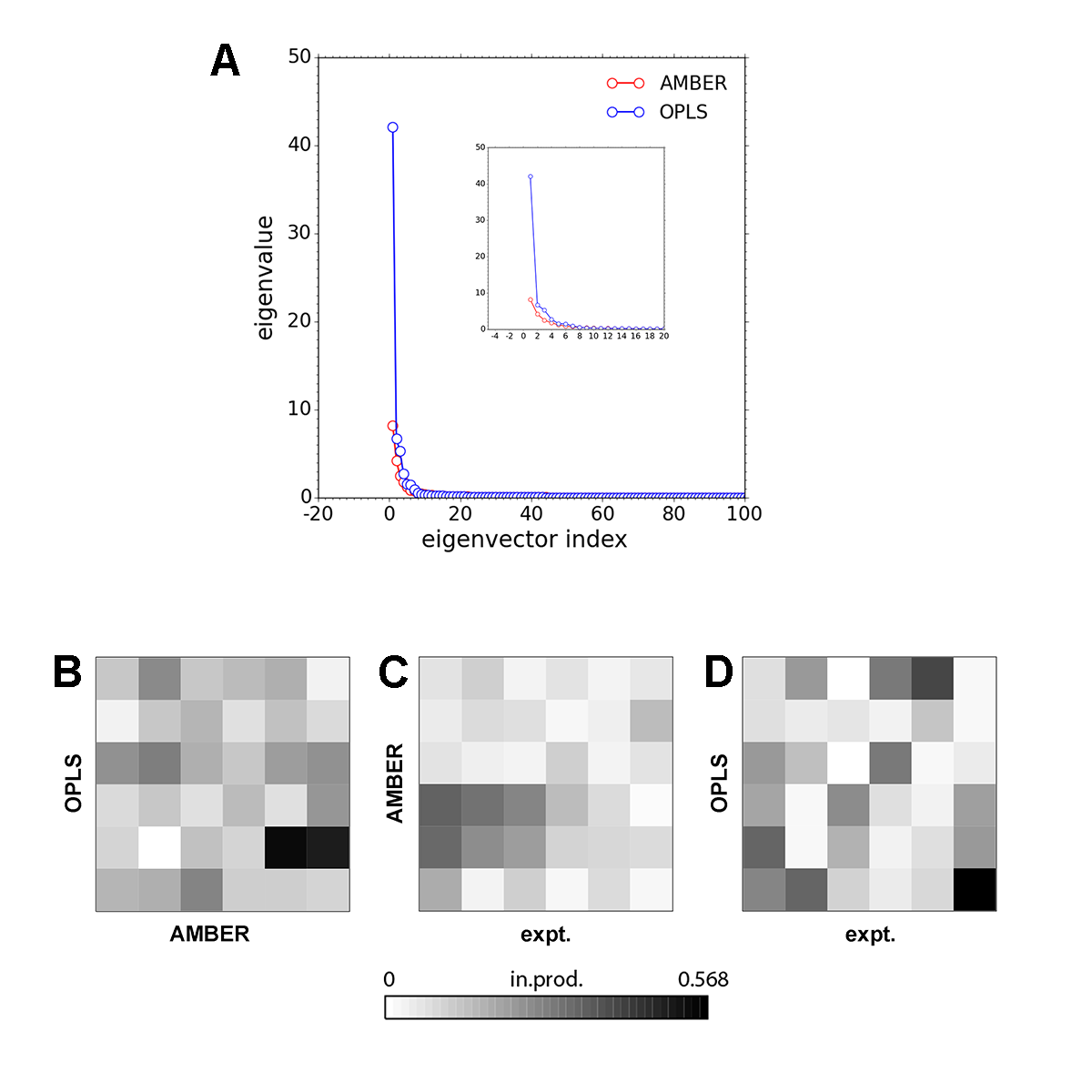


**Figure S7. Evaluation of PCA analysis.** (A) Eigenvalues for eigenvectors in the PCA analysis of AMBER and OPLS simulations. Simulation runs apo1, apo2, apo3, and bound are combined for the PCA analysis. Inside panel shows local details of the plot. (B)(C)(D) Comparison of inner products of top dominant eigenvectors from simulations and experiments. The experimental PCA were performed on an ensemble consisting of 44 full-length crystal structures of p38α. Note that only top 6 dominant eigenvectors of each system are shown here.


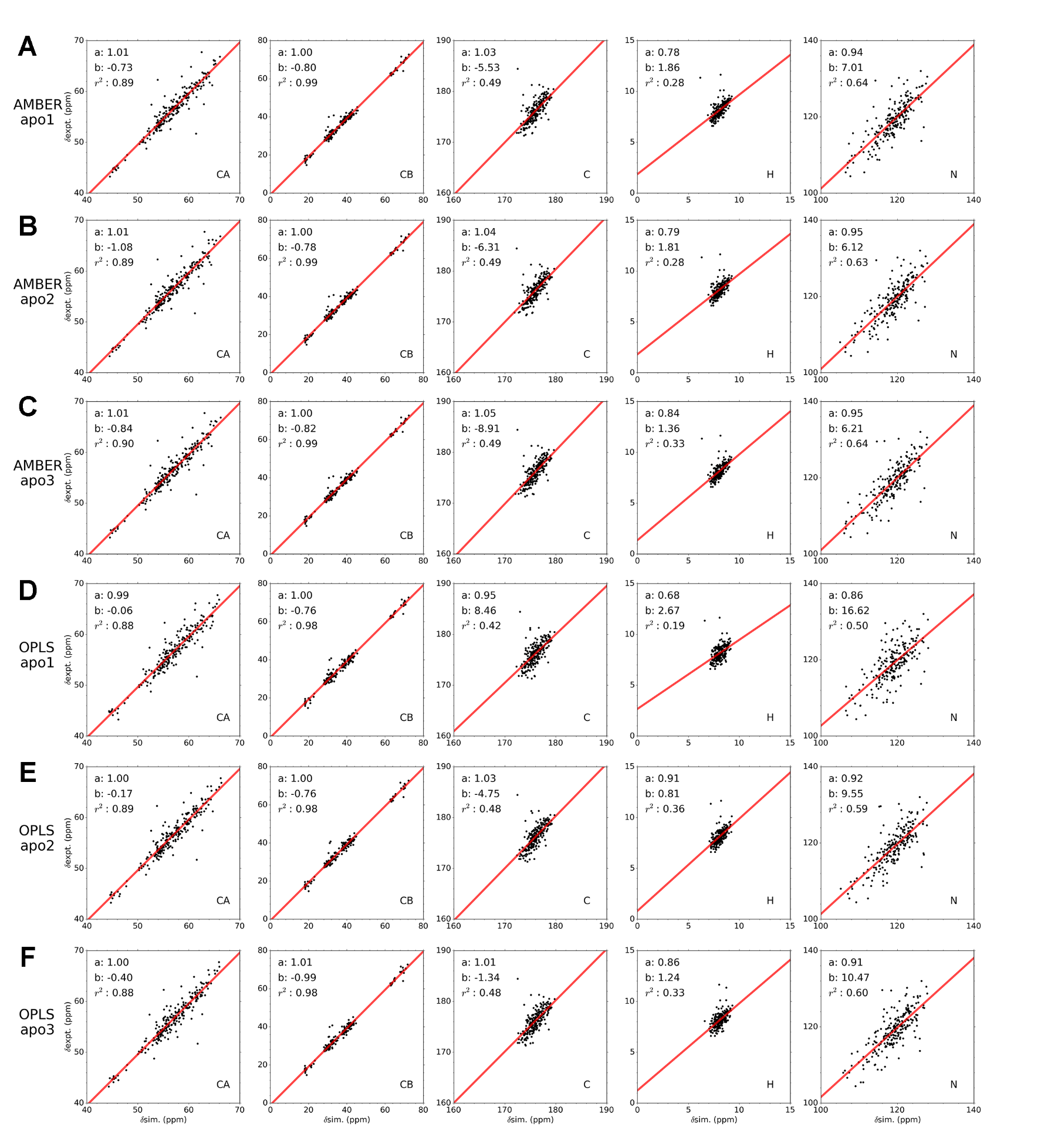


**Figure S8. Correlation of simulated chemical shifts calculated using PPM and experimental chemical shifts**. Results for AMBER (A)(B)(C) and OPLS (D)(E)(F) simulations are shown. The x-axis and y-axis of the correlation plot shows the simulated and experimental chemical shifts, respectively. The linear regression fit is shown as a red line, with slope a, intersection b, and correlation coefficient r^2^ shown in top left corner.


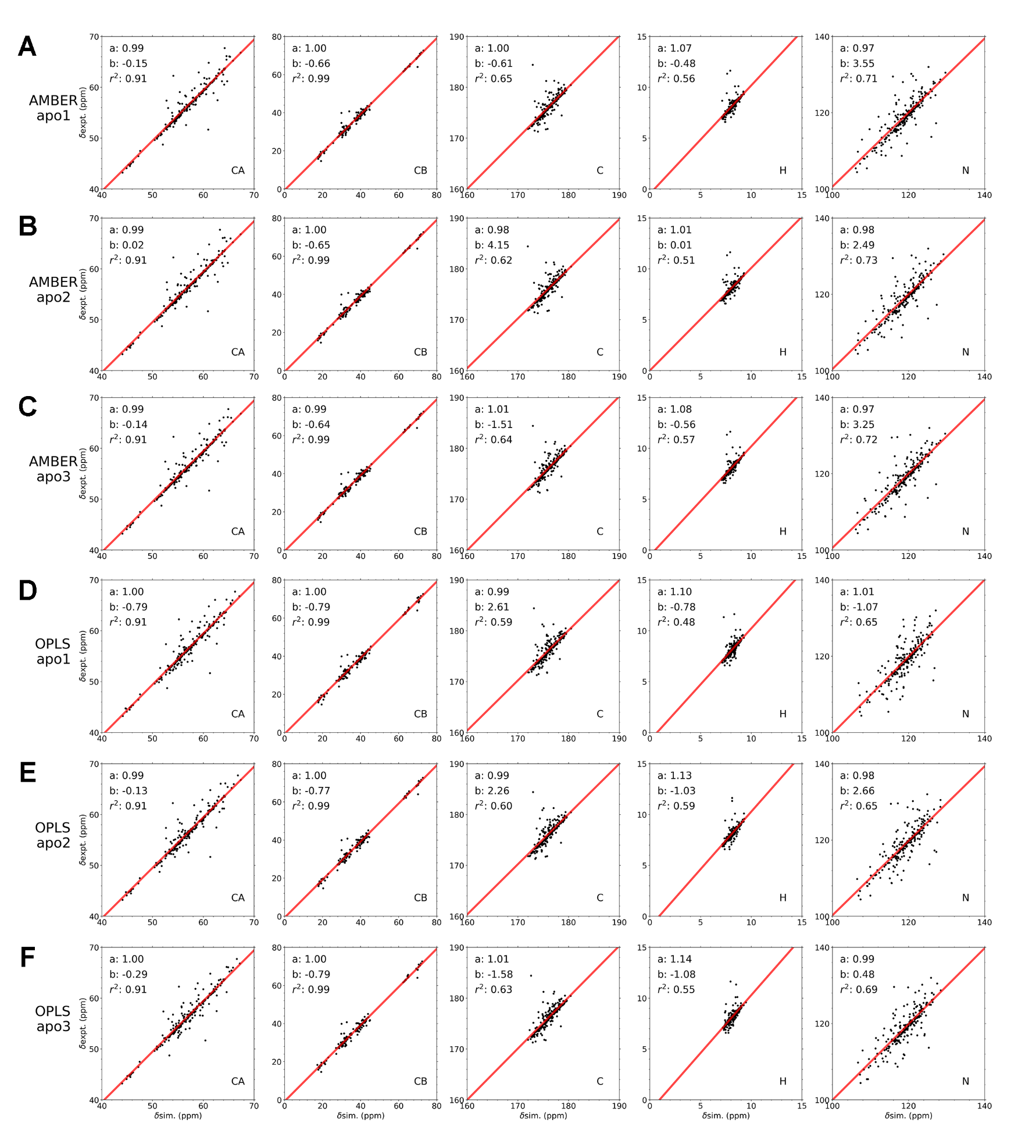


**Figure S9. Correlation of simulated chemical shifts calculated using SHIFTX2 and experimental chemical shifts**. Results for AMBER (A)(B)(C) and OPLS (D)(E)(F) simulations are shown. The x-axis and y-axis of the correlation plot shows the simulated and experimental chemical shifts, respectively. The linear regression fit is shown as a red line, with slope a, intersection b, and correlation coefficient r^2^ shown in top left corner.

**A**

**B**


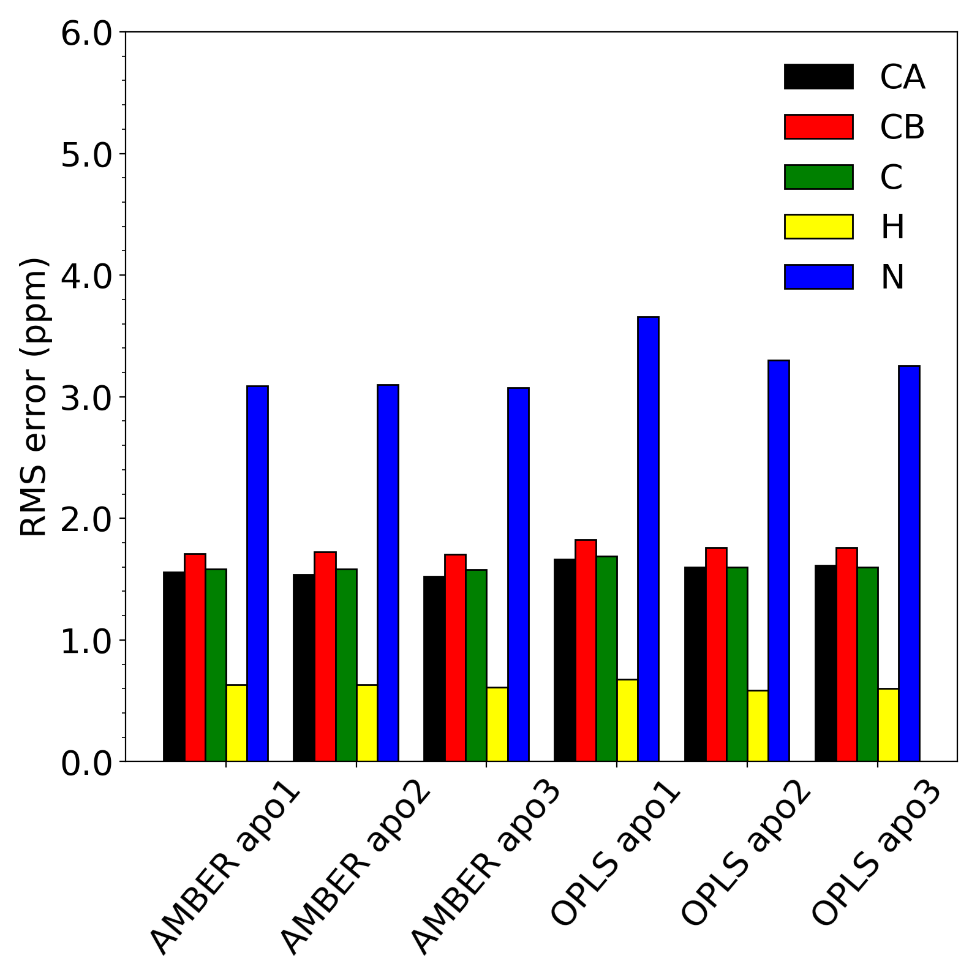

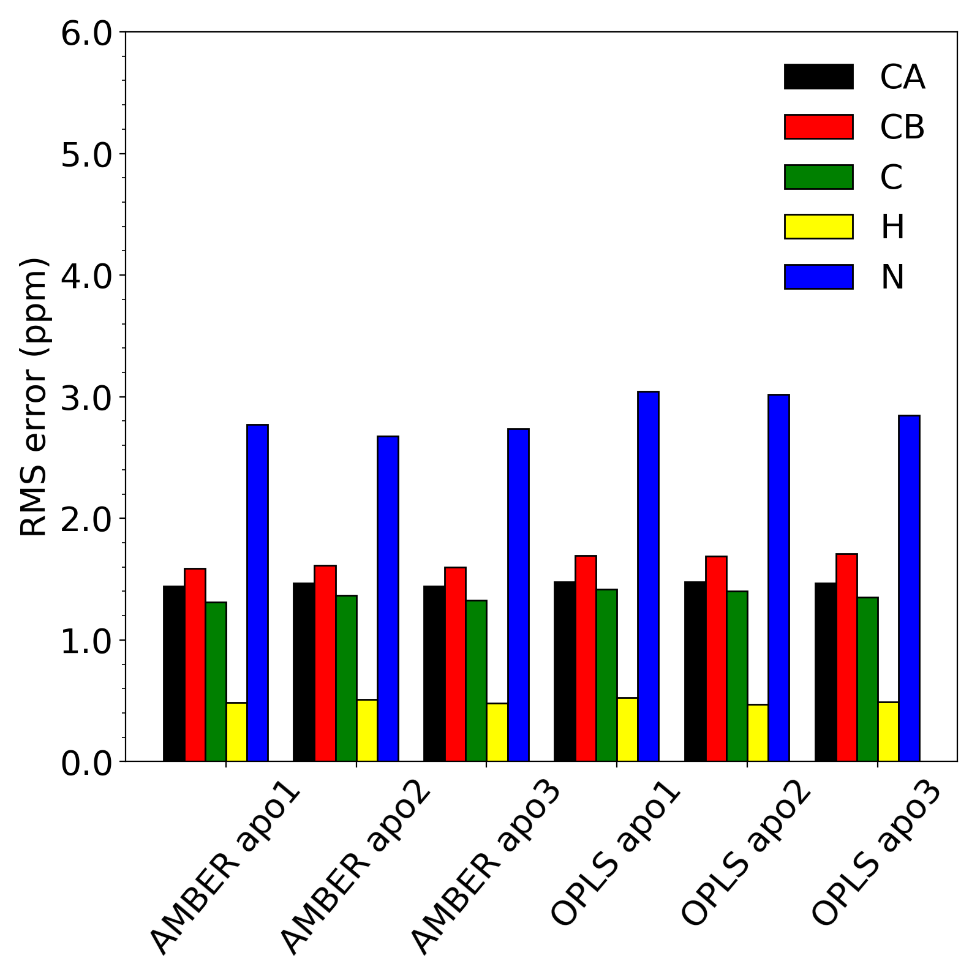


**Figure S10. RMS error between simulated and experimental chemical shifts of atom CA, CB, C, H, and N.** (A) RMS error for calculations using PPM. (B) RMS error for calculations using SHIFTX2.


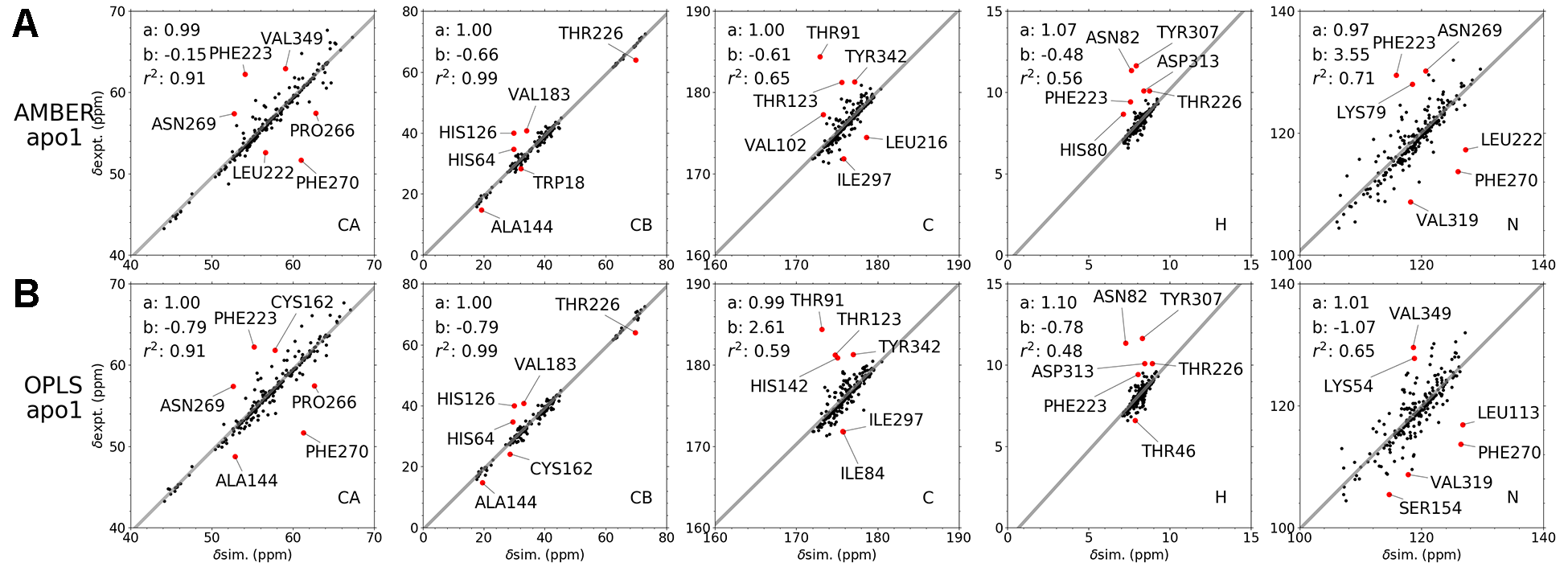


**Figure S11. Correlation of simulated and experimental chemical shifts.** Chemical shifts were calculated from MD snapshots at an interval of 1 ns using SHIFTX2. Results are shown for simulation runs AMBER apo1 (A) and OPLS apo1 (B). The x-axis and y-axis of the correlation plot are the simulated and experimental chemical shifts, respectively. The linear regression fit is shown as a gray line, with slope a, intersection b, and correlation coefficient r^2^ labeled in top left corner. The top six outlier residues with the largest RMS error are highlighted as red dots.


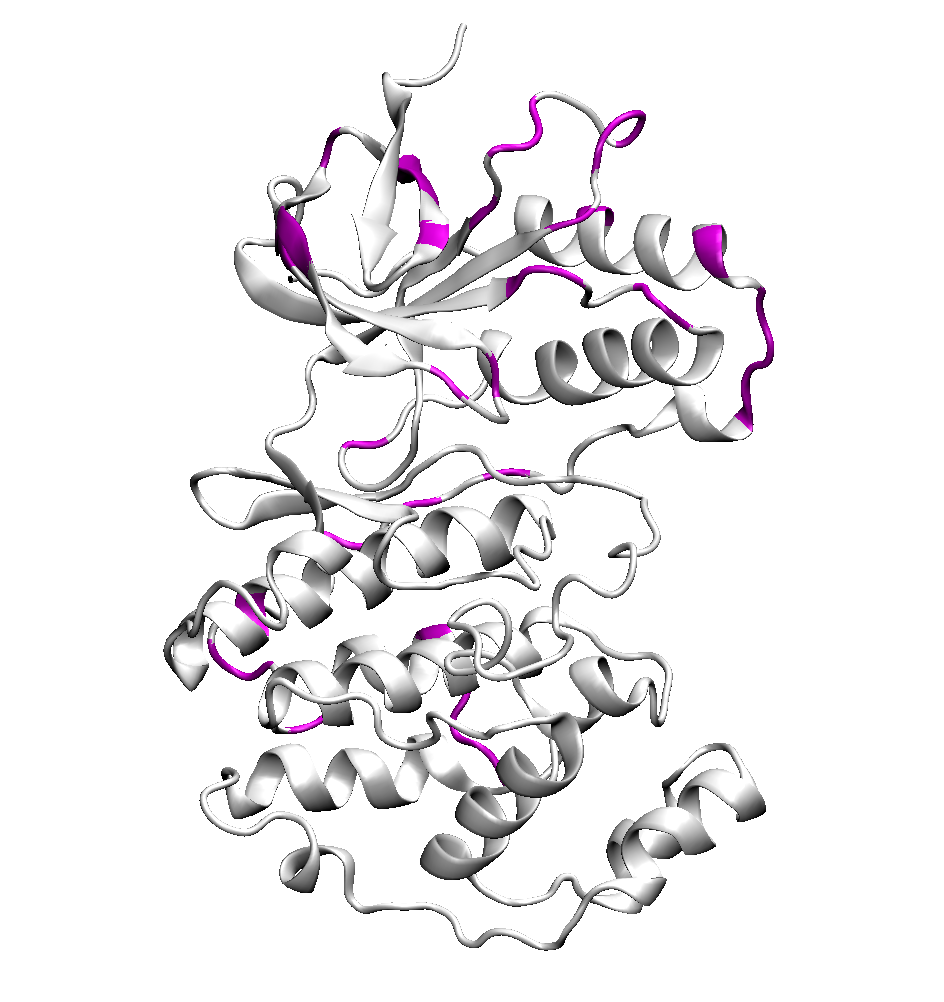


**A**


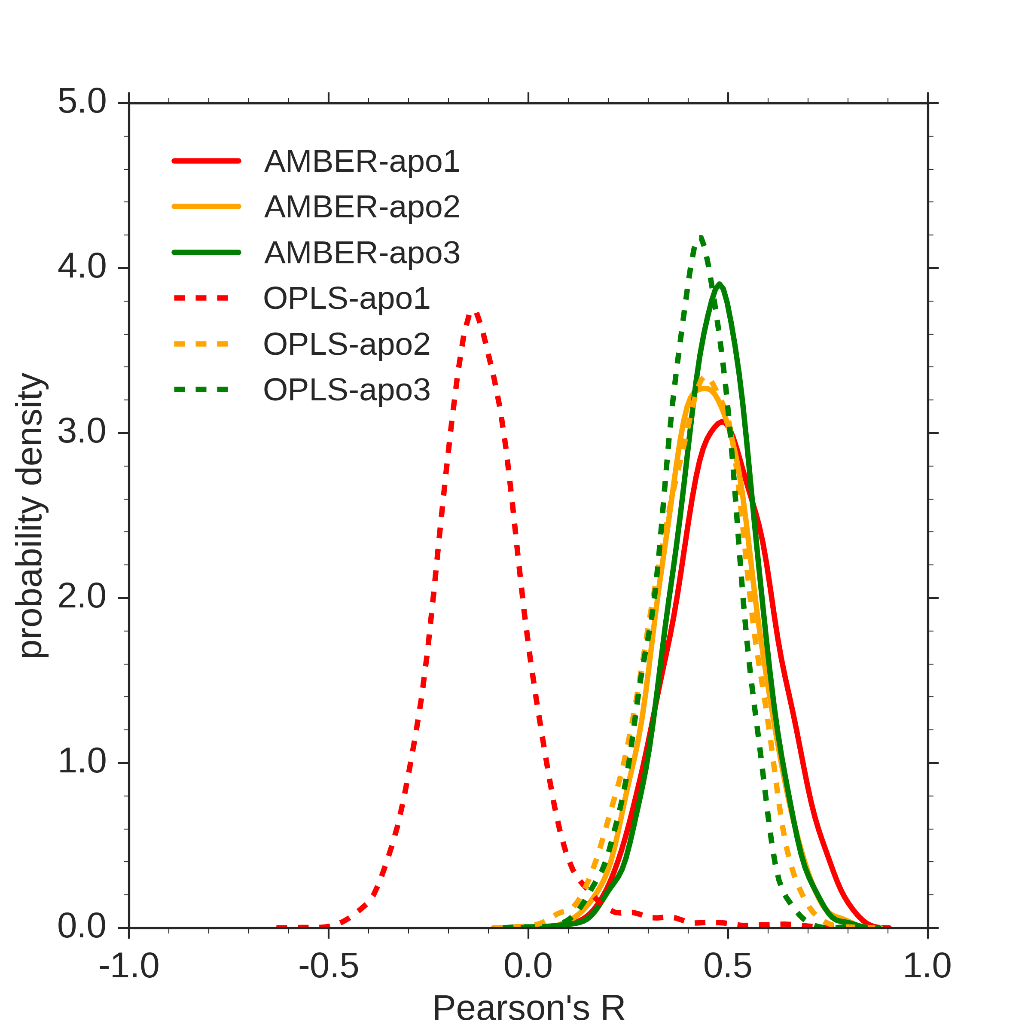


**B**

**Figure S12. Prediction of residual dipolar couplings from MD simulations.** (A) p38α structures in ribbon representation. Highlighted in magenta are the 39 residues that we use to obtain the correlation between simulated and experimental RDCs. (B) Probability density functions of Pearson’s R obtained from correlations between simulated and experimental RDCs.


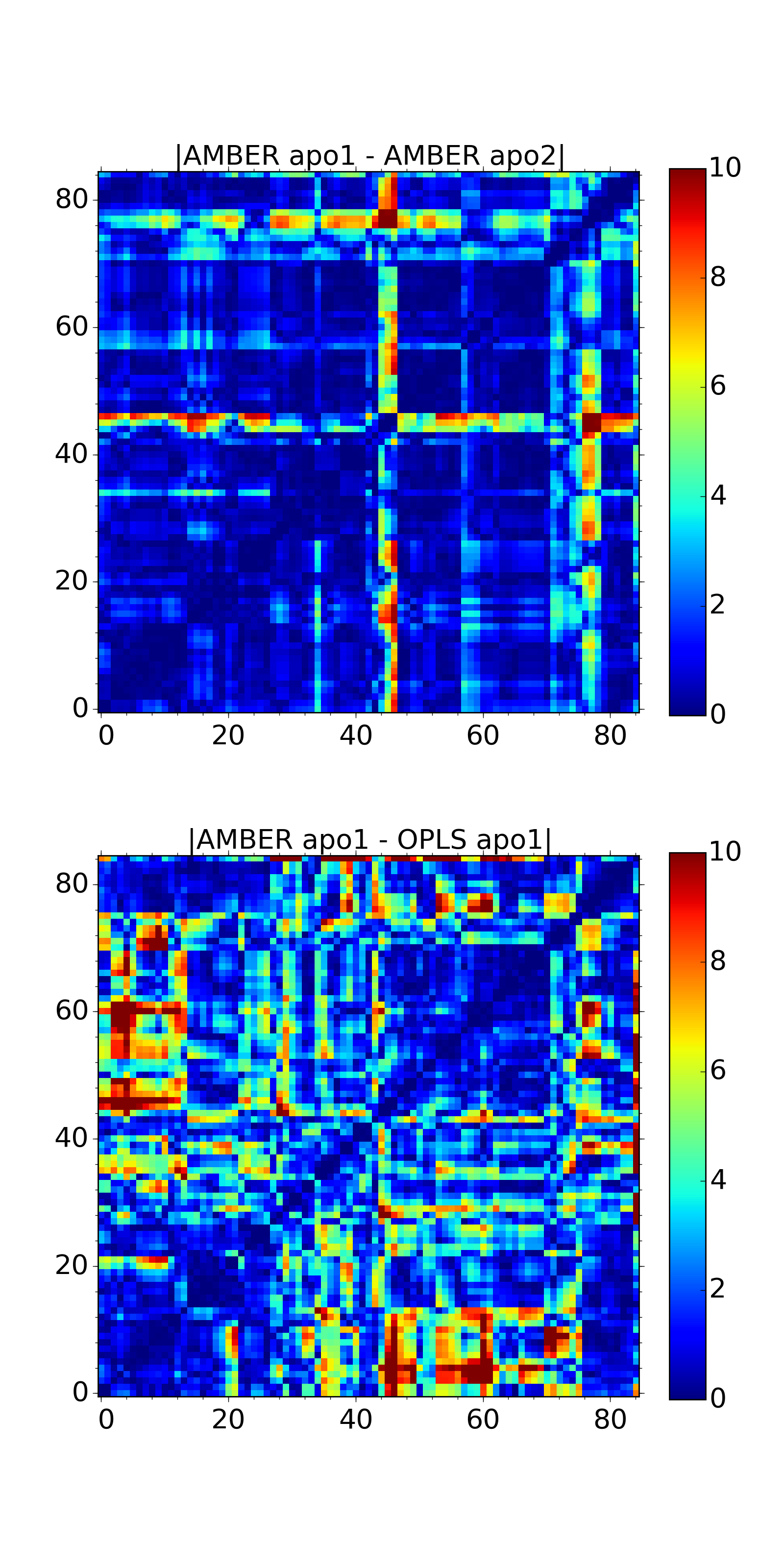


**A**

**B**

**Figure S13. Differences of charged residue distance matrix between different simulation runs.** (A) The difference between simulation run AMBER apo1 and AMBER apo2. (B) The difference between simulation run AMBER apo1 and OPLS apo1. Note that the absolute differences are shown here. Charged residues are listed in Table S6, including all ARG, LYS, ASP, and GLU residues.


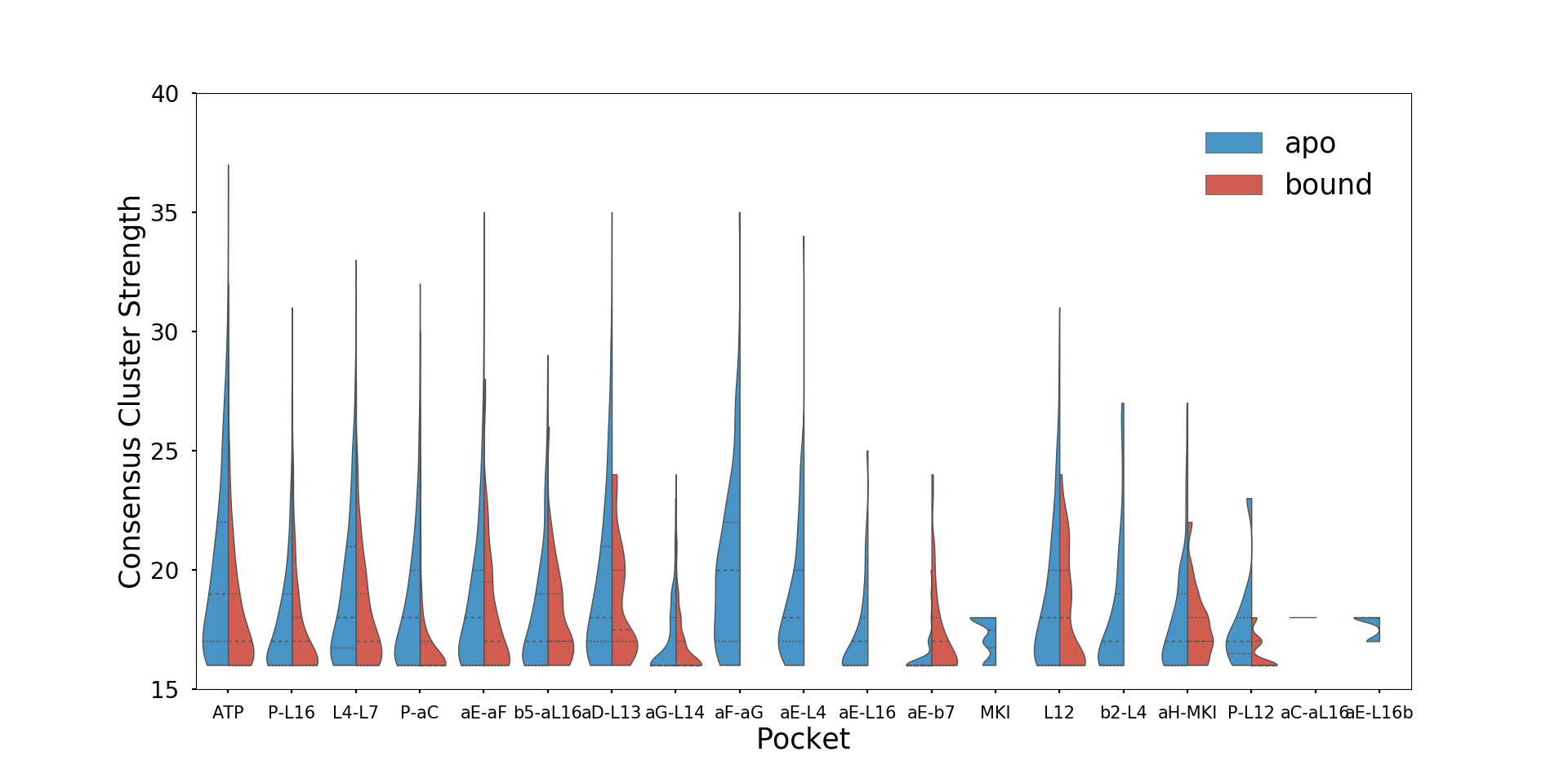

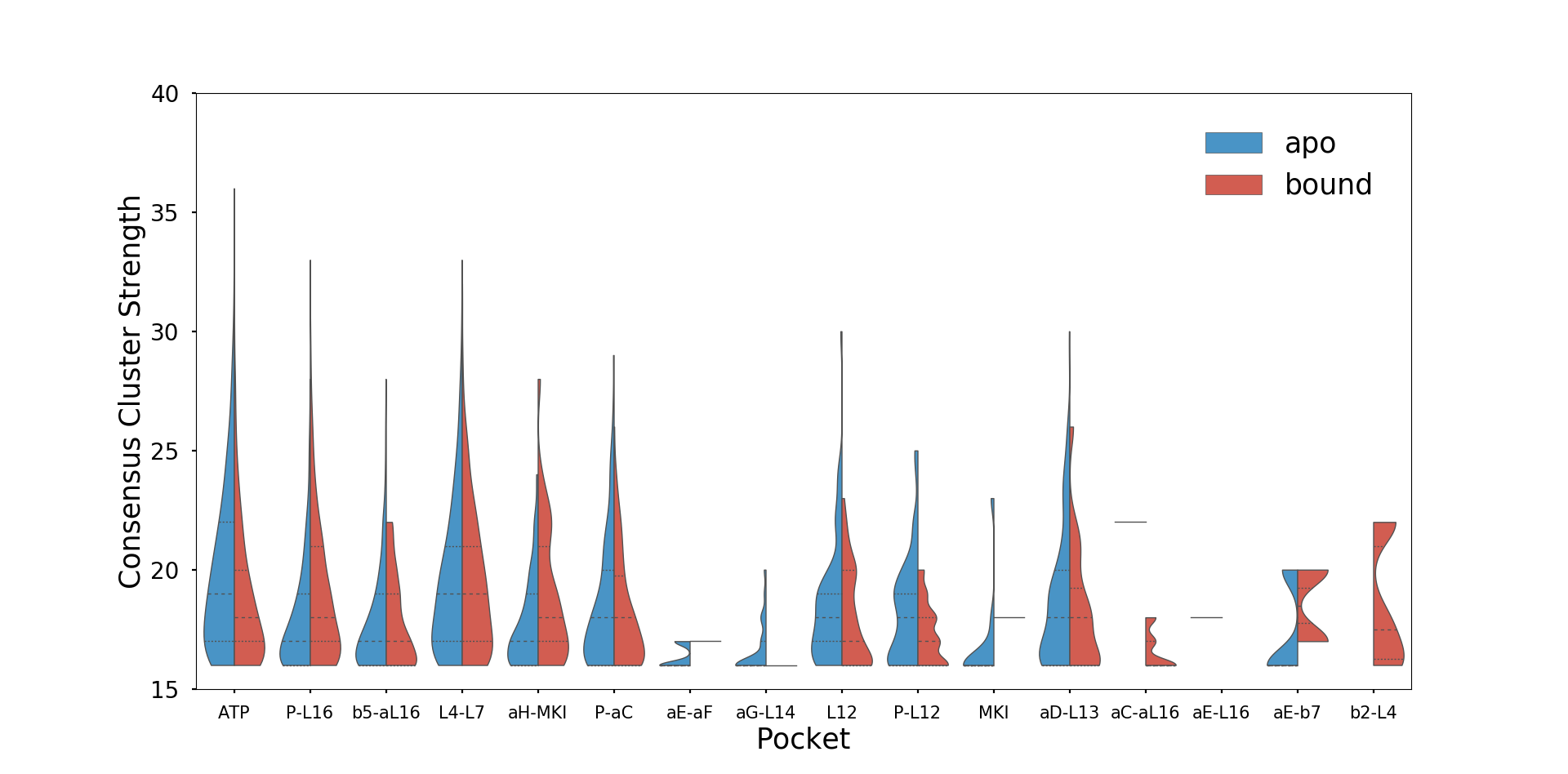


**A**

**B**

**Figure S14. Violin plot of consensus cluster strength for potential ligand-binding pockets identified from AMBER (A) and OPLS (B) simulations of apo and ligand-bound p38 **Results with consensus cluster strength S >= 16 are plotted with quartiles shown as dashed lines inside the violins. Data for apo and ligand-bound simulations were shown in blue and red violin, respectively.
